# Intravascular Food Reward

**DOI:** 10.1371/journal.pone.0024992

**Published:** 2011-09-27

**Authors:** Albino J. Oliveira-Maia, Craig D. Roberts, Q. David Walker, Brooke Luo, Cynthia Kuhn, Sidney A. Simon, Miguel A. L. Nicolelis

**Affiliations:** 1 Department of Neurobiology, Duke University Medical Center, Durham, North Carolina, United States of America; 2 Department of Pharmacology and Cancer Biology, Duke University Medical Center, Durham, North Carolina, United States of America; 3 Department of Biomedical Engineering, Duke University Medical Center, Durham, North Carolina, United States of America; 4 Department of Psychology and Neuroscience, Duke University Medical Center, Durham, North Carolina, United States of America; 5 Center for Neuroengineering, Duke University Medical Center, Durham, North Carolina, United States of America; 6 Edmond and Lily Safra International Institute of Neuroscience of Natal, Natal, Rio Grande do Norte, Brazil; University of Chicago, United States of America

## Abstract

Consumption of calorie-containing sugars elicits appetitive behavioral responses and dopamine release in the ventral striatum, even in the absence of sweet-taste transduction machinery. However, it is unclear if such reward-related postingestive effects reflect preabsorptive or postabsorptive events. In support of the importance of postabsorptive glucose detection, we found that, in rat behavioral tests, high concentration glucose solutions administered in the jugular vein were sufficient to condition a side-bias. Additionally, a lower concentration glucose solution conditioned robust behavioral responses when administered in the hepatic-portal, but not the jugular vein. Furthermore, enteric administration of glucose at a concentration that is sufficient to elicit behavioral conditioning resulted in a glycemic profile similar to that observed after administration of the low concentration glucose solution in the hepatic-portal, but not jugular vein. Finally using fast-scan cyclic voltammetry we found that, in accordance with behavioral findings, a low concentration glucose solution caused an increase in spontaneous dopamine release events in the nucleus accumbens shell when administered in the hepatic-portal, but not the jugular vein. These findings demonstrate that the postabsorptive effects of glucose are sufficient for the postingestive behavioral and dopaminergic reward-related responses that result from sugar consumption. Furthermore, glycemia levels in the hepatic-portal venous system contribute more significantly for this effect than systemic glycemia, arguing for the participation of an intra-abdominal visceral sensor for glucose.

## Introduction

The positive postingestive consequences of food consumption are a critical determinant of feeding behavior [1]. In fact, we recently found that in a two-bottle behavioral paradigm, even in animals in which functional sweet-taste transduction pathways are absent, oral consumption of sucrose (a calorie-containing saccharide), but not sucralose (a non-caloric artificial sweetener), was sufficient for the conditioning of robust side-preferences [2]. Furthermore, when such animals ingested sucrose, increases of dopamine release and neuronal activation were found in the nucleus accumbens (NAcc). Postingestive-dependent stimulation of the dopamine system has also been suggested in a human functional imaging study, in which sucralose and sucrose where presented at taste-intensity-paired concentrations, and only the latter activated dopaminergic midbrain areas [3]. The relevance of dopaminergic activation for postingestive-dependent learning has been confirmed in experiments conducted in rats, in which dopamine receptor antagonism in the NAcc inhibited the development of associations between non-caloric flavors and glucose injected directly into the duodenum [4].

With regard to the mechanisms leading to the behavioral reward-related effects of postingestive stimulation by sugars such as sucrose, the available data are not completely consistent. One study has indicated that direct stimulation of the intestinal mucosa is required [5], whereas others demonstrated the importance of postabsorptive effects by injecting nutrients intravenously [6], [7]. More recently it has also been suggested that, independently of taste, tissue glucose utilization is necessary for glucose consumption to cause dopamine release in the striatum [8]. In any case, the postingestive signaling pathways leading to behavioral conditioning and dopamine release remain controversial.

Here we were interested in clarifying the participation of preabsorptive and postabsorptive mechanisms in the reward-related effects of a calorie-containing sugar, independently of its taste. To that effect, we used rats to test the behavioral and dopaminergic responses to intravenous glucose administration in the jugular vein (JV) and hepatic-portal vein (HPV). We initially tested whether several different concentrations of glucose (5%, 22.5% and 50%), administered in the JV, were sufficient to condition a side-bias in a two-bottle preference test. In this experiment, saccharin was used as a control for potential intravascular taste stimulation. In another experiment, given the glycemic effects of the different glucose stimuli administered in the JV, the effects of 5% glucose administered in the HPV was tested in the same behavioral paradigm. In that experiment, mannitol was used as a control for osmotic effects. Subsequently, the glycemic effects of the different glucose stimuli used in these experiments were measured simultaneously in tail and HPV blood, to assess the relevance of changes in each of these locations for the behavioral effects observed previously. Finally, using cyclic voltammetry, dopamine transient frequency was measured in the NAcc and the effects of HPV and JV infusion of a 5% glucose solution was tested.

## Results

To clarify the participation of mucosal (intestinal) vs. postabsorptive mechanisms in taste-independent conditioning by postingestive effects in rats, we conducted experiments to test reversal of side-bias in a two-bottle behavioral paradigm [2]. Briefly, animals were tested for side-bias in a two-bottle water vs. water test under water and mild food restriction. To condition side-bias reversal, animals were then exposed to a 4 day conditioning protocol with free access to water given daily on alternate sides of the behavioral box. On the 2 days when animals were exposed to the side opposite to original bias, 3 mL glucose solution was administered simultaneously to water consumption and contingent upon licking behavior (3 µL/lick for the first 1000 water licks). On the remaining 2 days, when animals were exposed to the side of original bias, vehicle was given under the same conditions. In some animals, previously implanted with vascular catheters, conditioning stimuli (glucose or vehicle) were infused intravenously, while in others they were delivered orally through an independent cannula in the sipper tube used for water delivery (see Methods). In the final testing day, side-bias reversal was tested in all animals using a two-bottle water vs. water test (see Methods and Table 1 for details).

**Table 1:** Methods for Conditioning to Postingestive Effects.

			Oral Stimulus		Conditioning Stimulus
			Bias Side	Other Side	(intravenous or oral)
Day 1	Pre-conditioning test	Two-bottle	Water	Water	-
Day 2	Conditioning session 1	One-bottle	-	Water	Glucose or other
Day 3	Conditioning session 2	One-bottle	Water	-	Vehicle
Day 4	Conditioning session 3	One-bottle	-	Water	Glucose or other
Day 5	Conditioning session 4	One-bottle	Water	-	Vehicle
Day 6	Post-conditioning test	Two-bottle	Water	Water	-

**Figure 1:**
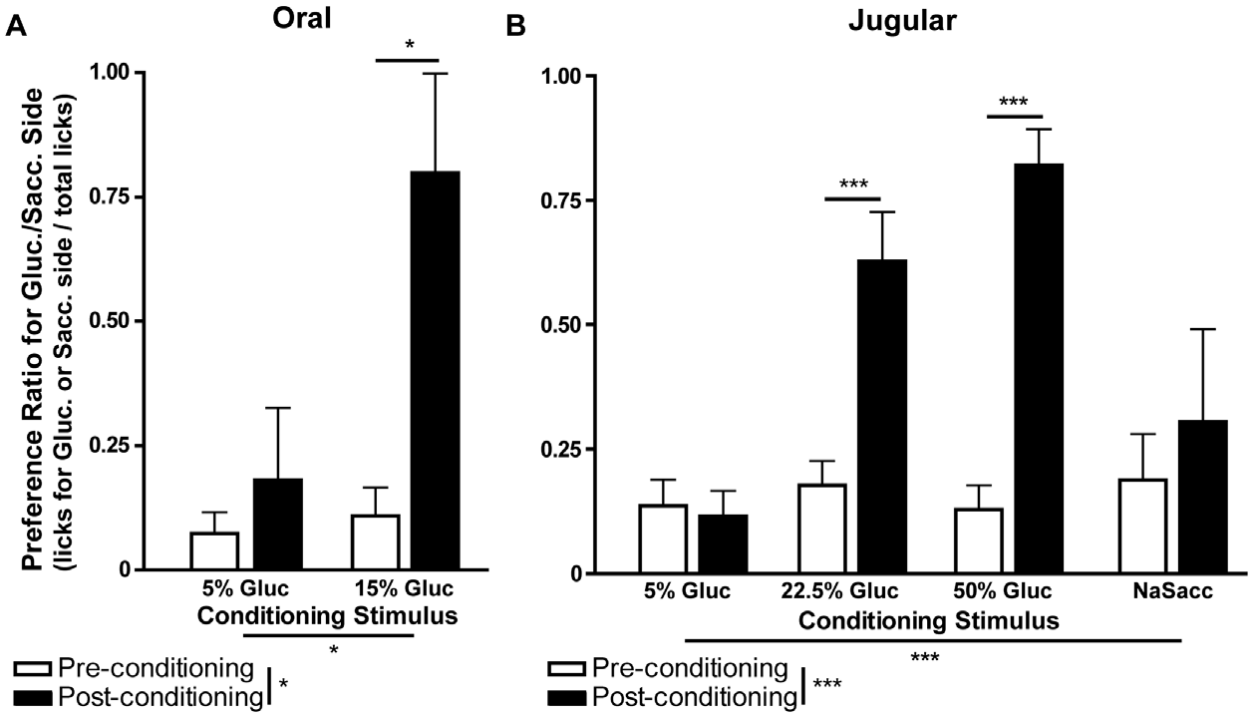
Jugular vein administration of glucose is sufficient to condition side-bias reversal. **A.** In animals conditioned with 15%, but not 5% glucose, delivered orally, there was a statistically significant reversal of original side-bias. **B.** In animals conditioned with stimuli administered in the JV, only those exposed to 22.5% and 50% glucose, but not 5% glucose or 3.16% NaSacc (to control for intravascular taste), reversed side-bias after conditioning. *p<0.05, **p<0.001, ***p<0.0001.

Two groups of animals were conditioned using either 5% (n = 6) or 15% (n = 5) glucose solutions delivered orally. Overall, the animals in these groups had higher consumption levels during vehicle conditioning sessions than during glucose sessions, an effect that could be due to the satiating effects of glucose administration or to the use of high levels of water deprivation (Fig. S1A). Side-bias reversal was tested by comparing preference for the side opposite to each animal's original bias in pre and post-conditioning two-bottle sessions. We found significant effects for conditioning stimulus (5% vs. 15%, F = 9.3, p = 0.014) and testing session (pre vs. post-conditioning, F = 7.9, p = 0.021), and a close to significant interaction between the two factors (F = 4.2, p = 0.071; repeated-measures two-way ANOVA). However, significant increases in pre vs. post-conditioning preference for the side associated to glucose delivery was found only in animals conditioned with 15% glucose (0.11±0.06 vs. 0.8±0.2, t = 3.3, p<0.05), but not those conditioned with 5% glucose (0.07±0.04 vs. 0.18±0.15, t = 0.6, p>0.05; post-hoc Bonferroni t-tests; Fig. 1A).

Using the same behavioral protocol, we tested the effects of JV infusions of low (5%, n = 10), intermediate (22.5%, n = 7) and high (50%, n = 11) glucose solutions. Sodium saccharin (NaSacc; 3.16%, shown previously to be the optimal concentration for intravascular stimulation of sweet taste [9]) was used as a control for potential intravascular taste stimulation (n = 5). Similarly to what was found in animals conditioned orally, overall these rats had higher consumption levels during vehicle conditioning sessions than during glucose or saccharin sessions (Fig. S1B). Regarding side-bias reversal, we found significant effects for conditioning stimulus (low vs. intermediate vs. high glucose vs. NaSacc, F = 7.8, p<0.001), testing session (pre vs. post-conditioning, F = 45.3, p<0.001) and the interaction between these factors (F = 15.8, p<0.001; repeated-measures two-way ANOVA). Side-bias reversal was found in animals conditioned with JV infusions of 22.5% (0.18±0.05 vs. 0.63±0.10, t = 4.7, p<0.001) and 50% glucose (0.13±0.05 vs. 0.82±0.07, t = 9.1, p<0.001), but not those conditioned with 5% glucose (0.14±0.05 vs. 0.12±0.05, t = 0.3, p>0.05) or NaSacc (0.19±0.09 vs. 0.3±0.19, t = 1, p>0.05; post-hoc Bonferroni t-tests; Fig. 1B). In summary, while the two higher concentration glucose solutions resulted in side-bias reversal, no effect was found for NaSacc, thus eliminating intravascular taste as the cue for these behavioral changes.

**Figure 2:**
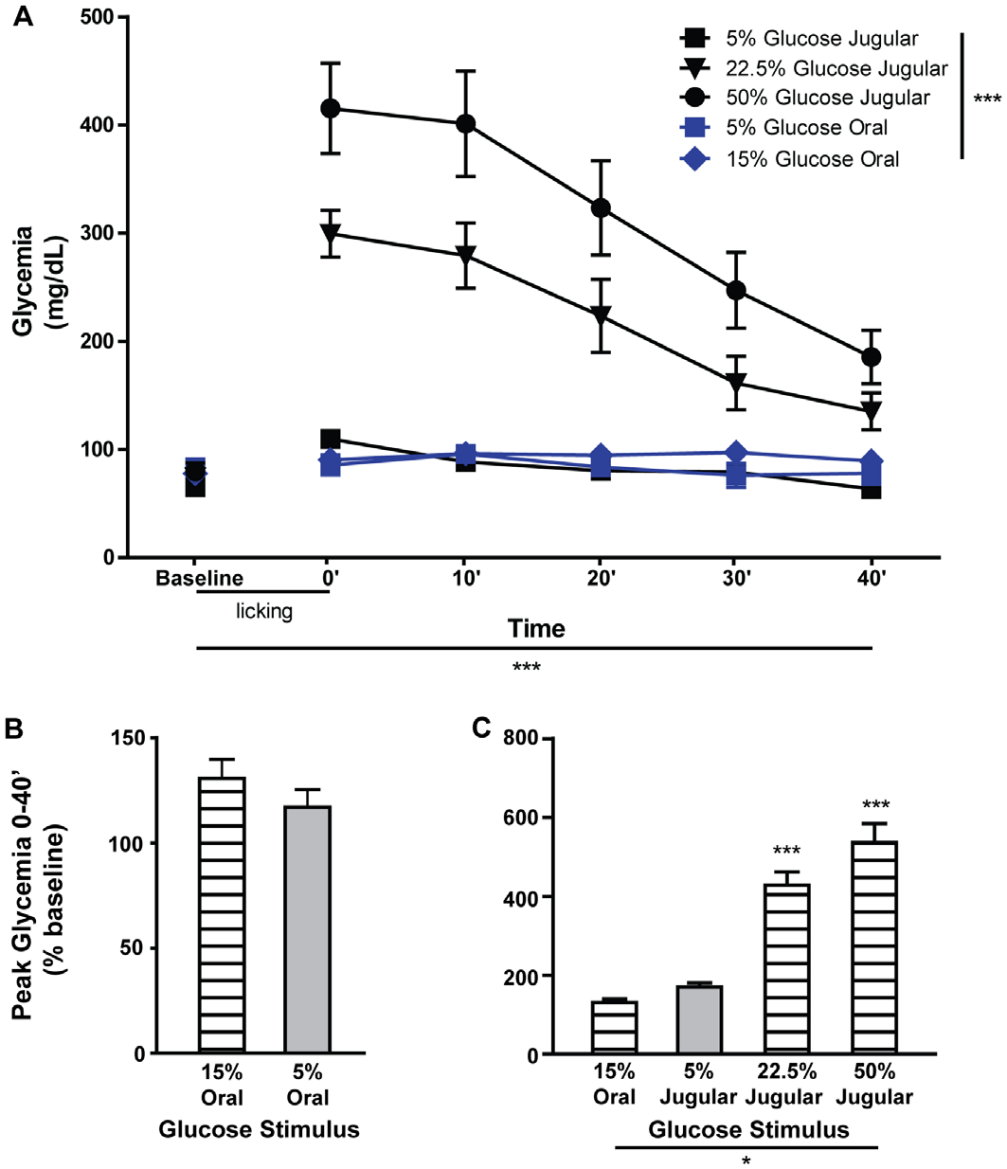
Jugular vein infusion of glucose stimuli that condition side-bias reversal also lead to extreme tail-blood hyperglycemia. **A.** In awake animals, glycemia (mg/dL) was measured from tail blood at the start (baseline), end (0′) and at 10 minute intervals after (10′–40′) behavioral sessions where one of the glucose solutions used for conditioning was administered. Significant overall effects were found for stimulus (5% vs. 22.5% vs. 50% JV glucose vs. 5% vs. 15% oral glucose; F = 25.5, p<0.0001), time (baseline vs. 0′ vs. 10′ vs. 20′ vs. 30′ vs. 40′; F = 38, p<0.0001) and the interaction between these factors (F = 12.6, p<0.0001; repeated-measures two-way ANOVA). Given this interaction, data was analyzed separately for each stimulus, showing a significant effect for time in all cases (JV 5% glucose, F = 14.5, p<0.0001; JV 22.5% glucose, F = 20, p<0.0001; JV 50% glucose, F = 28, p<0.0001; oral 15% glucose, F = 3.2, p = 0.036) with the exception of oral 5% glucose (F = 2.7, p = 0.087; repeated-measures one-way ANOVA). Furthermore, when glycemia at each time-point was compared to the respective baseline measure, significant effects were found at several time-points for JV 5% glucose (0′ and 10′), JV 22.5% glucose (0′, 10′, 20′ and 30′), JV 50% glucose (0′, 10′, 20′, 30′, and 40′), oral 15% glucose (10′, 20′ and 30′), but not for 5% oral glucose (post-hoc Bonferroni t-tests, see Table S1 for details). **B–C.** Peak relative glycemia (% of baseline) was compared after administration of glucose stimuli previously shown to be effective to condition side-bias reversal (hatched bars) and others that failed to do so (gray bars). We did not find significant differences between animals that consumed 5% or 15% glucose orally (B). Furthermore, glycemia after oral delivery of 15% glucose was significantly lower relative to that found after other stimuli that induced side-bias reversal (22.5% and 50%), but did not differ relative to 5% glucose, that did not condition such reversal (C). *p<0.05, ***p<0.0001.

To better understand the glycemic impact of the different glucose stimuli, separate groups of animals were allowed free access to water in 10 minute-long behavioral sessions while 3 mL of each of the glucose solutions used for conditioning were administered simultaneously to water consumption and contingent upon licking (3 µL/lick for the first 1000 water licks; 5% oral, n = 3; 15% oral, n = 4; 5% JV, n = 6; 22.5% JV, n = 4; 50% JV, n = 6). Glycemia was measured from tail blood before (baseline) and at several time-points after glucose administration (0′–40′). With the exception of oral 5% glucose, all stimuli caused a significant increase of peripheral glycemia (Fig. 2A, see Table S1 for data). This effect was also verified for JV 5% glucose, which did not condition side-bias reversal, showing that elevation of peripheral glycemia is insufficient to induce side bias changes. To further clarify the glycemic profile of each stimulus, both absolute (mg/dL) and relative measures (% baseline) of glycemia were analyzed in terms of mean and peak glycemia. In animals in which glucose was administered orally, no differences were found for peak relative glycemia (% baseline) after exposure to 5% and 15% glucose (117±8 vs. 131±9, respectively, t = 1.1, p = 0.33, unpaired t-test; Fig. 2B). When glycemia after 15% oral glucose, which was effective in conditioning side-bias reversal, was compared to those resulting from JV administration of glucose, a significant main effect was found (F = 37.7, p<0.0001, one-way ANOVA). Pair-wise comparisons revealed differences relative to JV 22.5% (428±34, t = 5.8, p<0.001) and 50% glucose (536±48, t = 8.6, p<0.001), but not relative to 5% glucose (170±10, t = 0.8, p>0.05; post-hoc Bonferroni t-tests; Fig. 2C). Similar results were found for peak and mean absolute glycemia (Figs. S2A, B, D and E) and mean relative glycemia (Figs. S3A and B).

**Figure 3:**
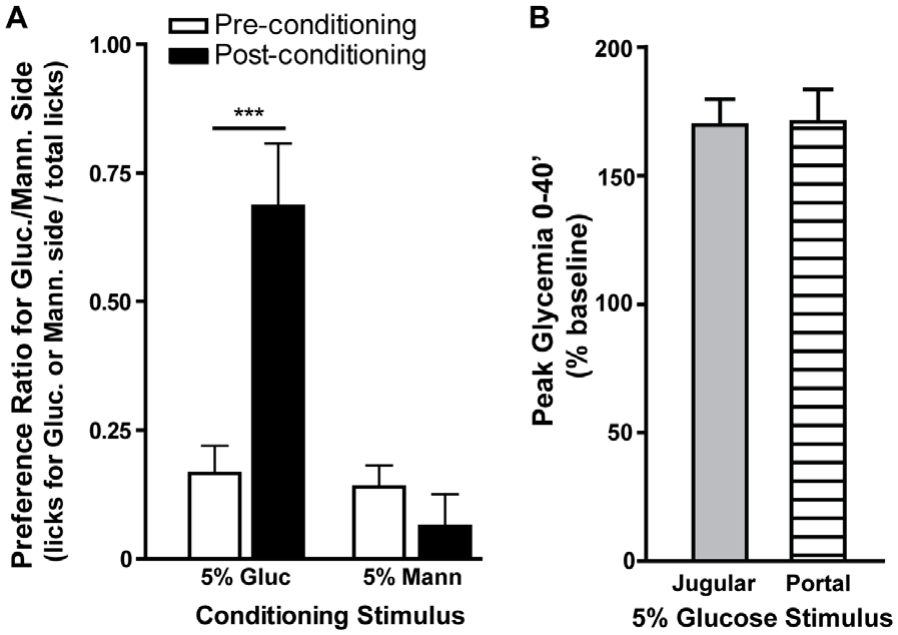
Hepatic-portal vein administration of glucose conditions side-bias reversal at lower concentrations than those needed with jugular vein administration. **A.** In animals conditioned with 5% glucose, but not 5% mannitol, administered in the HPV, we found a significant reversal of original side-bias. **B.** In awake animals, peak tail blood glycemia (% of baseline) after HPV 5% glucose, that conditioned side-bias reversal (hatched bar), was not significantly different from that observed after JV administration of 5% glucose, that failed to condition these behavioral effects (gray bar). ***p<0.0001.

Our findings suggest that, while intravenous glucose was sufficient to condition side-bias reversal in a two-bottle water vs. water test, this was only possible when glycemia levels much higher than those resulting from oral glucose were induced. We thus hypothesized that systemic hyperglycemia from JV glucose administration was necessary to drive a peripheral sensor that is activated following gastrointestinal glucose administration. The HPV system has an optimal location for such a sensor, since it carries most nutrient-rich blood from the gastrointestinal system, before it is released into the systemic circulation. To test this hypothesis, the same conditioning protocol was performed with 5% glucose administered through HPV catheters (n = 10), rather than orally or through the JV. As an osmotic control, another (sweet tasting) monosaccharide (5% mannitol [10]) was applied as a conditioning stimulus in an alternate group of animals (n = 5 Fig. S1C). In this experiment no differences in consumption were found during vehicle vs. glucose or mannitol conditioning sessions. Rather, animals conditioned with glucose had overall higher consumption levels than those conditioned with mannitol (Fig. S1C). When considering the behavioral effects in other conditioning sessions (Figs. S1A and S1B) this suggests that, under these conditions, mannitol produced a contextual aversive effect that generalized to vehicle sessions. Regarding side-bias reversal, significant effects were found for conditioning stimulus (glucose vs. mannitol, F = 10.2, p = 0.007), testing session (pre vs. post-conditioning, F = 5.2, p = 0.04) and the interaction between these factors (F = 9.5, p = 0.009; repeated-measures two-way ANOVA). Furthermore, in support of our hypothesis, a significant increase was found in pre vs. post-conditioning preference for the side associated with glucose (0.17±0.05 vs. 0.69±0.12, t = 4.7, p<0.001), but not mannitol infusion during conditioning (0.14±0.04 vs. 0.06±0.06, t = 0.5, p>0.05; post-hoc Bonferroni t-tests, Fig. 3A). Thus, 5% glucose was effective in conditioning side-bias reversal when administered in the HPV (Fig. 3A), but not the JV (Fig. 1B). This variation in the behavioral effects of 5% glucose did not result from differences in systemic glycemia depending on the administration site, since peak tail blood glycemia (% baseline) after HPV (171±13) and JV administration (Fig. 1D) were not significantly different (t = 0.07, p = 0.9, unpaired t-test; Fig. 3B; see table S1 for data). Differences were also not found for peak and mean absolute glycemia (Figs. S2C and F) and mean relative glycemia (Fig. S3D).

**Figure 4:**
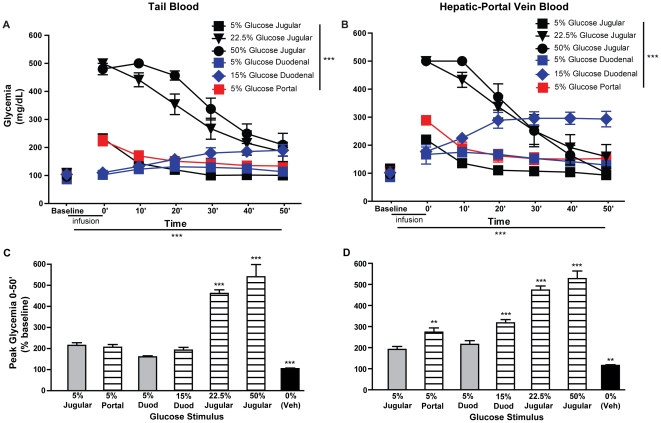
Increases in hepatic-portal vein blood glycemia parallel the capability of glucose stimuli to condition side-bias reversal. Tail and HPV blood glycemia was measured in anesthetized rats at the start (baseline) and several time-points after (0′–50′) perfusion of vehicle (duodenal water, JV saline or HPV saline) or one of the different glucose solutions used for conditioning (duodenal 5% or 15% glucose, JV 5%, 22.5% or 50% glucose or HPV 5% glucose). **A.** For *tail blood glycemia*, measurements after glucose injections, a significant overall effect was found for time, stimulus and the interaction between these factors (p<0.0001 for all; repeated-measures two-way ANOVA). Further comparisons were performed relative to JV 5% glucose, considered as a control stimulus that did not condition side-bias reversal, revealing several time-points after JV 22.5% and 50% and duodenal 15% glucose where glycemia was significantly elevated (see Table S2 for details). **B.** In terms of *HPV blood glycemia*, measurements after glucose injections, a significant overall effect was found for time, stimulus and their interaction (p<0.0001 for all; repeated-measures two-way ANOVA). Again, significant elevations of glycemia where found at several time-points after JV 22.5% and 50% and duodenal 15% glucose, when compared to those verified after JV 5% glucose (see Table S2 for details). **C–D.** Peak relative glycemia (% of baseline) was also compared after administration of vehicle (black bars), glucose stimuli previously shown to be effective to condition side-bias reversal (hatched bars) and others that failed to do so (gray bars). Peak glycemia for each stimulus was again compared to that after to JV 5% glucose, considered as a control stimulus that did not condition side-bias reversal and differences were parallel to the behavioral effects of each stimulus for HPV (D) but not tail (C) blood measurements. **p<0.001, ***p<0.0001.

Overall, these findings strongly indicate that an increase in HPV glycemia, rather than systemic glycemia, is a critical stimulus that results in taste-independent side-bias reversal. To further test this hypothesis, separate groups of anesthetized animals were injected with vehicle or the different glucose solutions used for conditioning, via catheters inserted into the duodenum (water, n = 5; 5% glucose, n = 4; 15% glucose, n = 6), JV (saline, n = 5; 5% glucose, n = 6; 22.5% glucose, n = 4; 50% glucose, n = 4) or HPV (saline, n = 4; 5% glucose, n = 7). Glycemia was measured from both tail and HPV blood at the start (baseline) and end (0′) of the stimulus perfusion, and every 10 minutes thereafter (10′–50′). Raw data were analyzed separately for vehicle and glucose injections and for tail and HPV blood glycemia (Fig. 4, see Table S2 for data). As is explained for glycemia in awake animals, peak and mean glycemia values were then compared (see data in Tables S3 and S4). For peak relative glycemia (% of baseline), a significant interaction was found between stimulus (JV vs. HPV vs. duodenal 5% glucose vs. duodenal 15% glucose vs. JV 22.5% glucose vs. JV 50% glucose vs. vehicle) and blood sampling site (tail vs. HPV; F = 11.4, p<0.001), allowing for separate analyses of tail and HPV glycemia (see Table S4 for details). In either case, glycemia after JV 5% glucose, considered as a control stimulus that did not condition side-bias reversal (see Fig. 1B), was compared to that observed after the remaining stimuli. For tail blood measurements, only JV 22.5% and 50% glucose and vehicle, but not HPV 5% and duodenal 5% and 15% glucose, differed significantly from JV 5% glucose (Fig. 4C). For HPV blood measurements however, duodenal 5% glucose, the only other glucose stimulus that did not condition side-bias reversal (see Fig. 1A), was also the only stimulus that did not differ significantly from JV 5% glucose (Fig. 4D). Thus, in accordance with our prior findings, HPV, but not systemic glycemia, as measured in anesthetized rats, accurately reflects side-bias reversal observed after conditioning in awake animals. This similarity between behavioral effects and HPV peak glycemic response was not found for mean glycemia (Table S4), suggesting that the relevant behavioral effects of glucose administration result from episodic high levels of glycemia rather than the effects of sustained glycemia changes. Similar results were found when absolute levels of glycemia (mg/dL) were compared (Table S3).

**Figure 5:**
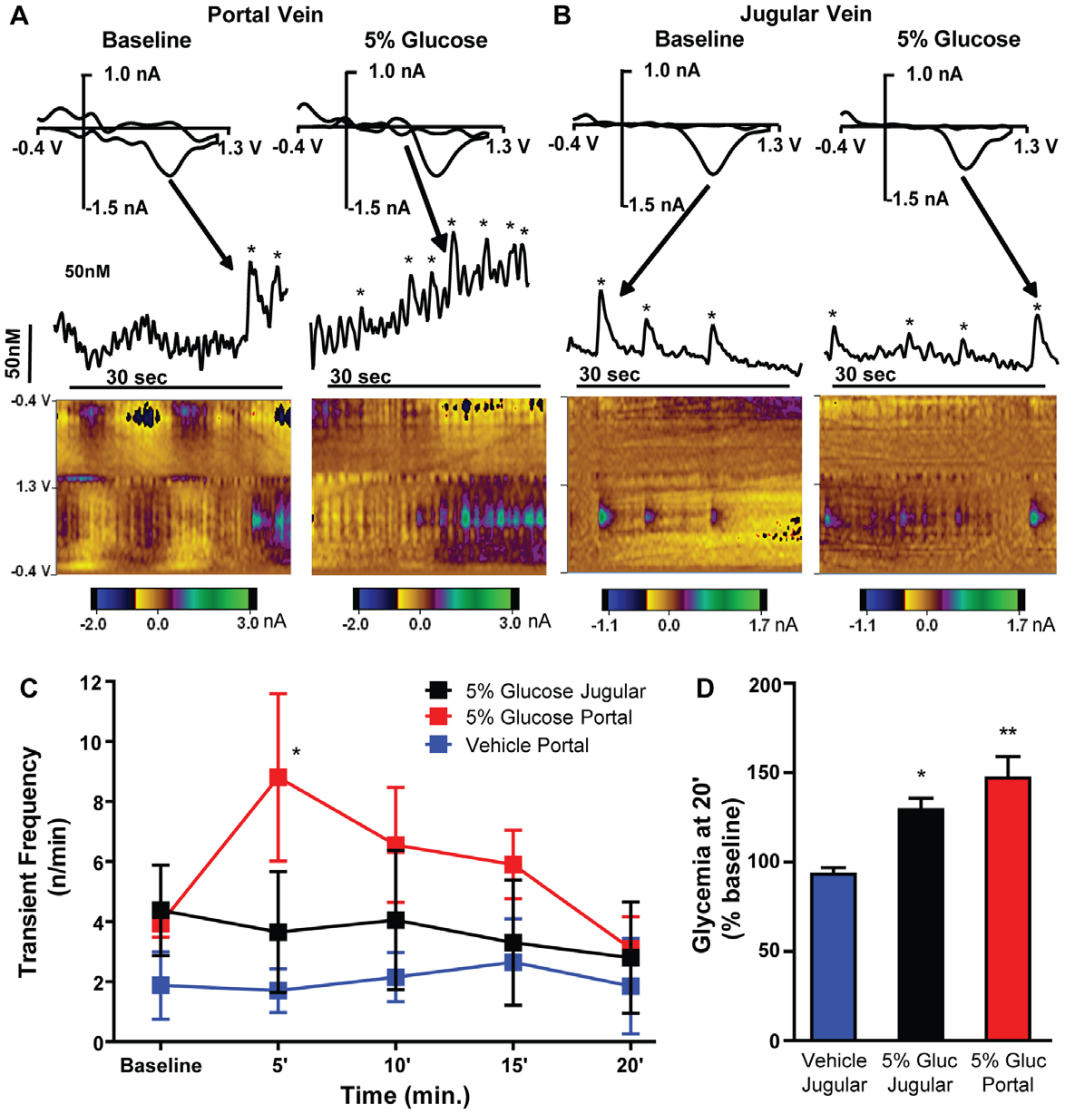
Nucleus accumbens dopamine transient frequency increases after injection of glucose in the hepatic-portal vein. Fast-scan cyclic voltammetry was used to identify spontaneous dopamine release events (transients) in the nucleus accumbens shell of anesthetized rats. Measurements were conducted for a baseline period, and also during and after infusion of 5% glucose in the JV (n = 4) or HPV (n = 4), or vehicle in the latter (n = 4). **A.** This panel shows example measurements in one animal before (figures on the left) and after (figures on the right) onset of 5% glucose infusion in the HPV. The two dimensional color plots at the bottom of the panel show the voltammetric current (encoded in color, nA) plotted against the applied potential on the **Y** axis (V) and the acquisition time on the **X** axis (sec.). The green spots at 0.62 V (the peak oxidation potential for dopamine) in the lower half of the plots represent dopamine oxidation at the surface of the carbon-fiber microelectrode. The current at the peak dopamine oxidation potentials was converted to dopamine concentration following post-calibration (see Methods) and is displayed above the color plots, approximately aligned with them in time. The panels near the top of the figure are cyclic voltammograms of the peaks indicated by arrows that were obtained by subtracting baseline voltammograms just prior to onset of the transient. Dopamine transients were considered as such when they had voltammograms that were significantly correlated (r>0.86) with those of a dopamine template elicited previously by electrical stimulation of the ventral tegmental area. The peaks that were thus classified as dopamine are indicated by asterisks. **B.** This panel shows an example measurement as was described above (A) but for JV, rather than HPV administration of 5% glucose. Note that the evoked activity is much less. **C.** Here we show dopamine transient frequency prior to infusion in each of the three groups (baseline) and in 5 min bins following infusion onset. Data were analyzed using two-way repeated measures ANOVA (see text) and post-hoc analysis indicated that, when compared to the respective time period for HPV vehicle administration, transient frequency in the HPV glucose group was significantly higher in the first 5 minutes after infusion onset (t = 3.1, p<0.05) but not in the remaining time periods (t<1.9, p>0.05 for all), or at any point in the JV glucose group (t<1.1, p>0.05 for all; post-hoc Bonferroni t-tests). **D.** In the same rats, tail blood glycemia was also measured prior to and 20 minutes after infusion of 5% glucose in the JV (n = 3) or HPV (n = 3), or vehicle in the latter (n = 4). No differences for absolute glycemia (mg/dL) were found between groups at baseline (PV vehicle - 157±7, JV 5% glucose - 169±22, HPV 5% glucose - 135±14; F = 1.4, p = 0.31, repeated-measures one-way ANOVA; t<1.7, p>0.05 for all pair-wise comparisons, post-hoc Bonferroni t-tests; data not shown in figure). However, as expected, an overall significant difference was found for relative glycemia (% baseline) at 20′ (F = 14.6, p = 0.0032, repeated-measures one-way ANOVA), with significant differences between glycemia after HPV vehicle (93±4) and that after JV 5% glucose (129±6; t = 3.5, p<0.05) and HPV 5% glucose (147±12; t = 5.2, p<0.01). Importantly, no differences were found between values after each of the two glucose stimuli (t = 1.6, p>0.05, post-hoc Bonferroni t-tests). *p<0.05, **p<0.001.

Prior studies have demonstrated that dopamine release can be evoked in the nucleus accumbens, in response to gastrointestinal delivery of sugars, independent of their taste [2], [4], [11]. We were therefore interested in understanding if dopamine release in this area would be differentially affected by JV vs. HPV administration of 5% glucose. Fast-scan cyclic voltammetry was used to identify spontaneous dopamine release events or “transients” [12], [13], [14], [15] in the nucleus accumbens shell of anesthetized rats (n = 12) (see methods). After obtaining baseline recordings for 6 to 8 minutes, rats were infused in the JV vein with 5% glucose or in the HPV with either 5% glucose or vehicle. The baseline transient frequency (3.4±0.7 transients/min), duration (520±68 msec) and amplitude (30±3 nM) were similar to what has been described in awake animals [12], [14], [15] (see Figs. 5A and B for representative recordings). Furthermore, once glucose induced changes were measured, cocaine (15 mg/kg) was administered to all animals to show the effects of dopamine reuptake inhibition under these conditions. We found that cocaine increased transient frequency and duration, without affecting amplitude (Fig. S5).

With regard to the changes in transient frequency after glucose administration, repeated-measures two-way ANOVA revealed a significant main effect for time (baseline vs. 1–5 min vs. 6–10 min vs. 11–15 min vs. 16–20 min; F = 2.7, p = 0.044) but not for treatment group (JV glucose vs. HPV glucose vs. HPV saline; F = 1.6, p = 0.26), and a significant interaction between factors (F = 2.4, p = 0.036, Fig. 5C). Given the significant interaction between factors, further comparisons for the effect of time were performed separately for each treatment group. For HPV saline and JV glucose groups, the effect of time was not significant (F = 0.5, p = 0.75 and F = 0.61, p = 0.66 respectively) and comparison at each time period relative to the respective baseline was also not significant (t<1.1 and t<1.5 respectively, p>0.05 for all). However, for the HPV glucose group, a significant effect of time was found (F = 3.9, p = 0.031; repeated-measures one-way ANOVA) and, when compared to baseline, the transient frequency was higher for the first 5 minutes after infusion onset (t = 3, p<0.05) but not in the remaining time periods (t<1.7, p>0.05 for all; post-hoc Bonferroni t-tests; see Table S5 for details). No effects were found for time, treatment group or their interaction with respect to transient duration or amplitude (Fig. S4). In these animals, tail blood glycemia was also determined before and 20 minutes after onset of JV or HPV infusions, showing, as expected, that the observed differences in transient frequency after glucose infusion are not due to differences in systemic glycemia (Fig. 5D). Thus, in accordance with the presented behavioral and glycemia data, at a lower concentration (5%), glucose significantly increased dopamine transient frequency when infused into the HPV, but not the JV.

## Discussion

In this study we demonstrate that postabsorptive mechanisms participate in the behavioral and dopaminergic reward-related effects of glucose, independently of the hedonically positive aspects of its taste. We first found that, in rats, high concentration glucose solutions administered in the JV were sufficient to condition a side-bias (Fig. 1B). However, this was only possible at glucose concentrations resulting in much higher glycemia than that resulting from oral consumption of glucose, at concentrations that were also sufficient to condition side-biases (Fig. 2). To clarify this inconsistency, further experiments were conducted to compare the effects of glucose administered in the JV and HPV, since the latter is the first site of glucose accumulation after absorption in the gut [16]. In this regard, we found that, at a lower concentration, glucose solutions conditioned robust side-biases when administered in the HPV, but not the JV (Fig. 3A). In support of this finding, enteric administration of glucose at a concentration that is sufficient to elicit behavioral conditioning (Fig. 1A) resulted in a glycemic profile similar to that observed after administration of the low concentration glucose solution in the HPV, but not the JV (Fig. 4). Finally, the administration of a low concentration glucose solution in the HPV, but not the JV, caused an increase in the frequency of dopamine transients in the nucleus accumbens shell, as measured by fast-scan cyclic voltammetry (Fig. 5). Overall, these findings support that the postabsorptive effects of glucose are sufficient for the postingestive behavioral and dopaminergic reward-related responses resulting from sugar consumption, and glycemia levels in the HPV system contribute more significantly to this effect than systemic glycemia.

The differential participation of oral and postingestive factors in the control of feeding behavior have been well characterized [1]. The postingestive controls of food intake were mostly recognized as inhibitory influences (i.e., satiation) but less research was dedicated to the positive postingestive effects of food intake [17]. While several early authors demonstrated that intragastric injection of nutrients can serve as an adequate behavioral reinforcer [18], [19], this finding was controversial and attributed to leaks in the gastric cannulation system [1], [20]. In fact, most authors developed work showing that nutrients injected directly into the stomach will condition a preference for an associated flavor [20], [21], [22], [23], [24], [25], and only recently did we find that, in mice without functional sweet-taste transduction in taste receptor cells, oral consumption of sucrose was sufficient for the conditioning of robust side-preferences, independently of any oral cues [2]. Here, the behavioral procedure developed previously in mice [2] was adapted for use in the rat, and we demonstrated that it could be used to measure behavioral conditioning resulting from enteric consumption of glucose (Fig. 1A).

While the literature on positive postingestive controls of food intake is now almost 60 years-old, the peripheral signals underlying these effects, particularly those of glucose, remain controversial [5]. Experiments with parenteral administration of glucose solutions in rats or rabbits, to test its effects as a signal for positive postingestive conditioning, have had both positive [7], [26], [27], [28], [29] and negative results [30], [31], [32]. Here we have demonstrated a dose-response effect in the capability of glucose to condition behavior, when administered in the JV (Fig. 1B). These results provide a reasonable explanation for prior discrepancies with JV glucose administration, since positive findings had been obtained with higher concentrations of glucose (30%) [7] than those used in experiments with negative results (10%) [31], [32].

In rats, the HPV was previously proposed as an important site for the detection of postabsorptive signals which support appetitive postingestive conditioning [6]. In a later study conducted in food deprived rats, HPV glucose injections were not found to condition flavor preferences, leading to the proposal that intestinal stimulation by nutrients is necessary for HPV infusions to condition behavior [5]. Here we find that, even in food deprived rats, HPV glucose infusion was sufficient to condition a side bias (Fig. 3A). When compared to the above noted negative experiments [5], these results cannot be explained by the use of higher glucose concentrations (5% used here vs. their 10%), but may be a consequence of differences in the rate of infusion (no less than 0.3 mL/min here vs. 0.083 mL/min). Furthermore, in accordance with previous findings [6], infusion of the same concentration of glucose in the JV had no such effects (Fig. 1B). Thus, while our data cannot exclude the importance of intestinal nutrient stimulation for appetitive postingestive conditioning, they clearly show that it is not necessary, and that postabsorptive factors, acting peripherally in the HPV system, are sufficient for such behavioral effects.

It is important to note that glucose and other nutrients present in the gut will be absorbed, resulting in a greater increase in blood nutrient levels in the HPV than in the systemic circulation [16]. This effect will presumably be additive to that of nutrient injection into the HPV, presenting a reasonable explanation for the previously noted association between the presence of nutrients in the intestine and the capacity of HPV infusions to condition behavior [5], [6]. Thus, when comparing the behavioral effects of enteric, JV and/or HPV nutrient infusions, it is important to directly measure blood nutrient levels resulting from these infusions. However, only a few of the studies investigating the positive conditioning effects of intravenous glucose administration have included systemic blood glycemia measurements, with mixed results [27], [31], [33], and none included these measurements in HPV blood. Here we show, both in awake and anesthetized rats, that tail blood glycemia measurements did not entirely explain the differential behavioral effects of enteric, JV and HPV glucose infusions (Figs. 2, 3B, 4A, 4C, S2 and S3; Tables S1, S2, S3 and S4). However, when measurements were performed in the HPV blood of anesthetized animals, peak glycemia was found to be a good correlate of the capacity of glucose solutions to condition side-biases (Figs. 4B and 4D; Tables S2, S3 and S4). This was not true for mean glycemia, reflecting the fact that intravenous injection of glucose did not result in an entirely physiological glycemic profile, and also suggesting that the observed behavioral effects of glucose administration were more dependent on the highest levels of glycemia than on sustained glycemia elevations. In any case, these findings further support the importance of postabsorptive glucose load in the HPV for appetitive postingestive conditioning showing, importantly, that this is true even when nutrients are administered in the gut, rather than directly in the JV or HPV.

Experiments performed to investigate the negative (i.e., satiating) effects of nutrients injected into the HPV have also been performed in several species. As described for positive conditioning, inconsistent results have been noted, (see [6], [34] for reviews). The reason for these differences has been variously attributed to the animal's nutritional status, the protocol for HPV infusion and the different testing paradigms [34]. In fact, most of these factors will presumably impact the nutrient level in the HPV, suggesting that this factor could underlie the satiating effects of intravenous nutrient infusions, as is described here for positive behavioral conditioning.

In the behavioral experiments with glucose infusions in the JV or HPV, control solutions were also used, to differentiate between effects due to the nutritional character of glucose, from those that could result from taste stimulation or changes in osmolality. For JV infusion experiments, a sodium saccharin solution was used, at a concentration that was previously shown to be optimal for intravascular stimulation of sweet taste [9]. For HPV infusions, mannitol was used since, at equal concentrations to glucose, this sweet-tasting monosaccharide causes similar changes in plasma osmolality, but has minimal metabolic effects [10]. Contrary to glucose, both saccharin and mannitol did not condition side-bias changes after infusion into the JV or HPV respectively, eliminating taste and plasma osmolality as the mechanism underlying those behavioral effects (Figs. 1B and 3A). These important controls supported our prior findings of taste-independent behavioral conditioning with sucrose in mice [2], and also the early reports of instrumental and place preference conditioning resulting from intragastric and intravenous administration of nutrients, in the absence of orosensory or olfactory cues [18], [19], [26], [27].

The role of dopamine in mediating the appetitive characteristics of food has previously been well established [35]. Elevation of dopamine levels in the nucleus accumbens (NAcc) occurs upon consumption of calorie-containing sugars such as sucrose [36], [37], and both sweet taste and postingestive stimulation have been shown to be sufficient for this effect [2], [38], [39]. However, the postingestive mechanisms leading to dopamine release in the NAcc after consumption of sugars, remain unclear. Arguing for the importance of preabsorptive stimulation, the gut-derived hormone ghrelin has been shown to stimulate mesoaccumbens dopamine release [40]. Evidence for postabsorptive controls of striatal dopamine release has also been presented, but the reported findings are somewhat equivocal [8], [41], [42]. Importantly, the effects of glycemia on striatal dopamine homeostasis has been attributed to glucosensing neurons in the brain [41], in particular since increases in dorsal striatum dopamine levels have been found to occur when glucose is infused directly in the substantia nigra [43]. Here, using cyclic voltammetry, we found that 5% glucose infused in the HPV, but not the JV, resulted in an increase of dopamine transient events (Figs. 5A–C), while causing a similar and physiologically relevant increase in peripheral glycemia in both cases (Figs. 3B, 4A, 4C and 5D). This finding confirms that postabsorptive glucose signals are sufficient to elicit dopamine release in the NAcc, also demonstrating, to our knowledge for the first time, that these postabsorptive effects are dependent on the increase of glycemia in the HPV, rather than from direct detection in the brain or alternate mechanisms, such as intravascular taste. The differential dopaminergic effects of 5% glucose administered in the JV and HPV are particularly relevant given that they are parallel to the differential effects of these treatments on behavior (Figs. 1B and 3A) and HPV glycemia (Figs. 4B and 4D), suggesting that the latter may be the underlying explanatory factor for both behavioral and neurochemical effects of glucose administration.

Although spontaneous dopamine transients have been reported in awake rats [44], [45], we note that, to our knowledge, this is the first report in anesthetized rats (Fig. 5A and 5B). Nevertheless, we found that all three characteristics used to describe transients (frequency, duration and amplitude) are quite similar to those reported in awake rats [14], [15], [44], [46], [47]. Furthermore, even in anesthetized rats, transient frequency was responsive to both glucose infusion into the HPV (Fig. 5C) and to i.p. administration of cocaine (Fig. S5A). Prior experiments using cyclic voltammetry had described NAcc dopaminergic responses to intra-oral sucrose [47] or saccharin [39] infusion. In comparison with these previous voltammetric studies, we report sustained increases in transient frequency over a period of minutes after intravenous glucose infusion, rather than the responses over a few seconds which occur after oral stimulus presentation [46], [48]. Similarly to what has been previously described for nomifensine [15] and cocaine [45] administration, we also found that injection of 15 mg/kg cocaine caused an increase of NAcc dopamine transient frequency (Fig. S5A) and duration (Fig. S5B). However, we found no effects for transient amplitude (Fig. S5C). Thus, the effects of psychostimulants are similar in awake and anesthetized rats. Investigating effects on transients in anesthetized rodents may be most appropriate in contexts in which environment and conditioning are unwanted confounds.

The NAcc, where we measured dopamine transients, is not a homogenous structure. There are two main subdivisions, the Nacc core and Nacc shell, for which functional differences in reward processing and dopamine release kinetics have been reported [49], [50]. The voltammetry data reported here was measured with electrodes implanted in the shell subregion. In fact, the effects we observed after cocaine injection (Fig. S5A) are in accordance with previous findings, showing that cocaine increases transient frequency in this subregion, but not the NAcc core [51]. Dopamine transmission within the NAcc shell has been proposed to participate in primary reward processing in response to both psychostimulants [49], [51] and oral ingestive stimuli [39], while the release of dopamine in the NAcc core is thought to have a more specific role in the response to reward predicting cues in the context of learned associations [39], [49]. We now show that HPV glucose infusion causes an increase of dopamine transient frequency in the NAcc shell, an effect that, as suggested from the effects of dopamine receptor antagonism in the same area, could be relevant for flavor preference learning induced by the post-oral consequences of carbohydrates [4]. In any case, further research is needed to explore the participation of the NAcc core in the postabsorptive responses to sugars.

Prior research had suggested that sweet-detection and transduction mechanisms that are found on taste cells on the tongue, are also present in the gut, and are a critical postingestive regulator of the appetite for sugars [52]. However, we and others have shown that non-nutritive sweeteners, that will also activate these mechanisms [52], do not condition side [2] or flavor [53] preferences. Furthermore, even though taste-like detection and transduction mechanisms are defective, both Trpm5 KO [2] and T1R3 KO [53] mice will develop preferences conditioned by nutritive sugars. The results described here support that a postabsorptive factor, dependent on glycemic changes in the HPV, rather than systemically, contributes to the positive behavioral effects and the NAcc dopaminergic effects resulting from the consumption of sugars. While these results argue against a major role for the participation of a preabsorptive mechanism, such a mechanism cannot be excluded and, in fact, there is evidence that mucosal and postabsorptive factors could cooperate in postingestive conditioning [5]. In any case, the nature of glucose-dependent HPV postabsorptive factors is still to be determined. One possibility is the participation of insulin, particularly since this hormone was shown to be relevant for the dopaminergic response to hyperglycemia [41]. Another candidate intervening mechanism is net hepatic glucose uptake, since it is dependent on insulin levels [54] and is increased when glucose is delivered in the HPV, in comparison to peripheral infusion [55]. A relationship between liver energy status, measured in terms of hepatocyte ATP concentration, and food intake has also been shown [56]. Ultimately, these factors could also explain recent findings, showing that glucose utilization is necessary for the behavioral conditioning and dopaminergic effects of glucose consumption [8]. The mechanism for transmission of signals from the liver to the brain is also unclear. One hypothesis is neural transmission, possibly via glucose-sensitive vagal afferent neurons [57]. However, lesioning abdominal vagal and non-vagal neural afferents has not been shown to block flavor preferences conditioned by enteral administration of glucose polymers [58], [59], [60]. Another alternative, which has been less explored [61], is that humoral factors, possibly produced by the liver and/or pancreas, are released in the bloodstream and act directly in the brain to modulate food intake [62].

In summary, these findings support the claim that the postabsorptive effects of glucose are sufficient to induce appetitive behavioral responses towards sugar consumption, and that glycemia levels in the HPV system contribute more significantly for this effect than systemic glycemia. Moreover, these effects occur independently of taste, postingestive mucosal stimulation, and plasma osmolality. Furthermore, this is the first demonstration, that HPV glucose administration is sufficient to induce striatal dopamine transients, an effect that could underlie the positive behavioral effects of this treatment. Given the dramatic behavioral effects of gastrointestinal bypass surgery for weight reduction [63], [64], with potential effects on HPV nutrient levels, we believe that the mechanisms described here could play a part in the etiology and treatment of human obesity, thus meriting further and more extensive research in both animal and human models.

## Materials and Methods

### Ethics Statement

All procedures were carried out in strict accordance with protocols approved by the Duke University Institutional Animal Care and Use Committee (protocol number A329-07-12).

### Subjects

One-hundred forty-four male Long-Evans rats and 12 male Sprague-Dawley rats were obtained from Charles Rivers Laboratories (Raleigh, NC) and housed individually in Plexiglas cages. All animals were maintained on a 12 hr light/dark schedule and experiments were carried out in the light portion of the cycle. At the time of each experiment, animals were 3 to 6 months old and naïve for the stimuli used. Preliminary experiments demonstrated that, under the conditions necessary to conduct experiments with intravenous injections, stable licking rates could be obtained only when oral access to water was restricted to behavioral sessions. Thus, Purina rodent chow and water was available *ad libitum*, except for the duration of behavioral testing, when animals were food deprived overnight and had access to water only during the behavioral task. In experiments conducted in anesthetized animals, they were food and water restricted approximately 24 hours prior to the experiment. In those animals whose body weight dropped below 85% of baseline, food and water restriction was discontinued and the animals were excluded from the experiment.

### Stimuli

All infused solutions (d-glucose – 5%, 15%, 22.5% and 50%; d-mannitol – 5%; sodium saccharin – 3.16%) were prepared daily in deionized water or 0.9% NaCl and maintained at room temperature. Deionized water and 0.9% NaCl solution were also used and are designated as ‘vehicle’. Deionized water was also delivered orally, as were, in another set of experiments, 5% and 15% glucose solutions prepared in deionized water. d-glucose, d-mannitol, sodium saccharin, deionized water and NaCl are referred to in the paper as glucose, mannitol, saccharin, water and saline respectively. All chemicals were obtained from Sigma-Aldrich (St. Louis, MO) and were reagent-grade.

### Jugular and Hepatic Portal Vein (HPV) Catheterization

Polyurethane catheters (Strategic Applications, Inc., Libertyville, IL), were implanted in the right jugular vein in 75 rats and in the hepatic portal vein in 35 rats. Some animals were catheterized offsite (Charles Rivers Laboratories, Wilmington, MA) while others were catheterized in-house using the same procedure. Briefly, animals were anesthetized using 5% halothane followed by intraperitoneal or intramuscular injection of xylazine (5–20 mg/kg) and ketamine (75–100 mg/kg). Supplemental doses were administered whenever necessary. For jugular catheterization, a small incision was performed to expose the right jugular vein. The cranial end of the vein was ligated and a loose ligature was placed caudally to isolate a 5 mm section of the vessel. For portal catheterization, an abdominal midline incision was made, the cecum was pulled out and the mesenteric vein was identified. Similarly, the distal end of the vein was ligated and a loose ligature was placed proximally to isolate a section of the vessel. In either case, a catheter was inserted into an incision made between ligatures and secured in place by tying the loose ligature around the catheterized vessel. A small incision was then made in the scapular region and the catheter was subcutaneously tunneled and exteriorized through this incision. Patency was tested and the catheter was filled with a ‘locking solution’ (500 IU/mL heparin in 50% glucose) and sealed with a plug. A subcutaneous skin pocket was made cranially and the excess length of catheter was inserted into the pocket. Finally, the vessel incision site and scapular area skin incision were closed with wound clips. Animals were allowed to recover for 3 to 5 days after surgery before initiation of further experimental procedures. Before these procedures, the catheter locking solution was replaced by 50 IU/mL heparin in 0.9% NaCl solution, which was also used to maintain patency in behavioral experiments that were conducted across several days.

### Behavioral Setup

All behavioral tests were conducted in Med Associates (Med Associates Inc., St. Albans, VT) behavior boxes, each enclosed in a ventilated and sound-attenuating chamber, as described previously [65]. Briefly, chambers were equipped with two slots for sipper tubes in one of the walls. Access to sipper tubes could be blocked by computer-controlled doors and sipper slots were equipped with beam lickometers used for licking detection (Med Associates Inc., St. Albans, VT). Each sipper tube contained two 20-gauge stainless-steel cannulae that were connected to 50-ml chromatography columns (Kontes Flex-Columns; Fisher Scientific, Hampton, NH) where stimulus solutions were contained. The solution containers were housed outside the sound-attenuating chambers and kept at an elevated position to promote liquid flow by gravity. Computer-controlled solenoids (Parker Hannifin Corporation, Fairfield, NJ) regulated the flow of fluid such that within 10 ms after a lick was detected, one of the valves opened and delivered ∼3 µL of fluid. A syringe pump (Med Associates Inc., St. Albans, VT) was used to allow intravascular injection of solutions during behavioral tasks. The pump was activated 10 ms after a lick was detected, resulting in a ∼3 µL injection of whatever solution was contained in the syringe attached to the pump. Thus, the infusion rate was matched to licking rate (∼7 Hz) and the volume of fluid ingested was approximately equal to the amount infused.

### Conditioning to Postingestive Effects

#### General Protocol

To test if rats develop side-biases conditioned by glucose in a two-bottle paradigm, 72 animals were exposed to a conditioning protocol similar to what we used previously in mice [2]. For each animal, side-bias was determined in a **10 minute-long** test with water delivered on both sides of the behavioral box (two-bottle water vs. water test). Once a clear side bias was established, the animals were exposed for 4 days to **30 minute-long** sessions of free access to water presented daily on alternate sides of the box (one-bottle forced-choice training sessions). On the first and third days, water was presented on the side opposite to the initial side-bias and 3 mL of a conditioning stimulus was delivered simultaneously to water for the first 1000 licks (i.e., 1000×3 µL = 3 mL). On the second and fourth days, water was presented on the initial side-bias and 3 mL of vehicle (saline or water, see below for details) was delivered simultaneously to water for the first 1000 licks. Typically, the first 1000 licks occurred in up to 8 minutes. After training, reversal of side-bias was tested in **10 minute-long** two-bottle water vs. water tests. In these experiments, 8 animals did not complete the conditioning protocol due to disease or excessive weight loss and in 5 others there was an error in the conditioning protocol. Data from these 13 animals was excluded from analysis.

#### Conditioning Stimuli

In 11 animals conditioning was performed with vehicle (water) and glucose solutions (6 rats with 5% and 5 rats with 15%), delivered orally during licking for water. In 33 animals conditioning was performed with infusions of vehicle (water or saline – no differences were found according to vehicle subtype, data not shown) and saccharin (5 rats) or glucose solutions (10 rats with 5%, 7 rats with 22.5% and 11 rats with 50%) delivered through **jugular vein** catheters. In 15 animals conditioning was performed with infusions of 5% mannitol (5 rats) or 5% glucose (10 rats) delivered through **portal vein** catheters. In those animals where conditioning was performed using venous catheters, prior to both testing and conditioning sessions, the animal was anesthetized using 5% isoflurane and the catheter was connected to a syringe that was placed on the infusion pump.

### Blood Glucose Measurements

#### Awake Animals

Rats were initially trained to drink in daily 10 minute-long sessions of free access to water. Once stable consumption levels were obtained, a test session was conducted where, for the first 1000 water licks, a particular stimulus (∼3 mL) was delivered orally or infused, simultaneously to licking. In 7 animals glucose solutions (3 rats with 5% and 4 rats with 15%) were delivered orally. In 20 animals glucose solutions were delivered through jugular vein catheters (6 rats with 5%, 4 rats with 22.5% and 6 rats with 50%) or portal vein catheters (4 rats with 5%). On the test day, tail blood was collected for determination of glycemia both before and immediately after exposure to the testing chamber (respectively baseline and 0′), and also at 10 minute intervals after the animal was returned to his home-cage (10′, 20′, 30′, 40′). Glycemia was measured using a hand-held glucometer (Precision Xtra, Abbott Laboratories, Abbott Park, IL, USA; maximal detection level of 500 mg/dL).

#### Anesthetized Animals

Naïve rats were anesthetized using 5% halothane followed by intramuscular injection of 50 mg/kg pentobarbital, which has been shown to be one of the anesthetic strategies with small effects on glycemia [66]. Supplemental doses were administered whenever necessary. An abdominal midline incision was made. In 15 rats, that had not received any prior surgery, the stomach wall was punctured and a polyethylene catheter was inserted such that its' extremity was in the duodenum, approximately 2 cm from the pylorus. The remaining 30 rats had been previously implanted with jugular or portal vein catheters. Tail blood glucose levels were measured at 5 to 10 minute intervals. Once tail blood glycemia stabilized, a baseline glycemia measurement (0′) was recorded for both tail and portal vein blood. For collection of portal blood, the hepatic portal vein was identified close to the hepatic hilus and a 31 gauge needle was used to collect 0.05 to 0.1 mL of blood. 3 mL of a particular stimulus was then injected continuously across 8 minutes using a syringe pump (Med Associates Inc., St. Albans, VT), to emulate conditions during behavioral training sessions. Of the animals with duodenal catheters, 5 rats were injected with vehicle (water), 4 with 5% glucose and 6 with 15% glucose. In the 11 rats with portal vein catheters either vehicle (saline; n = 4) or 5% glucose was administered (n = 7). The remaining 19 rats had jugular vein catheters and received either vehicle (saline; n = 5) or glucose solutions (5%, n = 6; 22.5%, n = 4; 50%, n = 4). Tail blood and portal vein blood were subsequently collected at 10 minute intervals for up to 1 hour, starting 10 minutes after the initiation of infusions (10′, 20′, 30′, 40′, 50′ and 60′). Glycemia in tail and portal vein blood was measured as described above.

### Fast-scan cyclic voltammetry

#### Electrodes

Carbon-fiber microelectrodes were prepared from 7 µm diameter T-300 carbon fibers (Amoco, Greenville, SC) as previously described [67]. When necessary, epoxy was added to the glass/fiber interface to improve the seal. Approximately 50–100 µm of fiber was left exposed past the glass/carbon seal. All electrodes were soaked in 2-propanol with activated Norit-A charcoal for at least 10 min before use. After lowering to the starting depth in the nucleus accumbens, electrodes were conditioned by cycling for 15 minutes at 60 Hz with a triangle waveform (−0.4 V to 1.3 V vs Ag/AgCl at 400 V/sec) and then for another 15 min at the frequency used for data collection, 10 Hz [68]. Chloridized silver wires were used as reference electrodes and all potentials were reported vs Ag/AgCl.

#### Electrochemistry

For fast-scan cyclic voltammetry (FSCV) we used a UEI electrochemical instrument (University of North Carolina Department of Chemistry Electronics Facility, Chapel Hill, NC). National Instruments boards (PCI-6052 and PCI-6711E) and TH-1 software (ESA, Chelmsford, MA) were used for waveform generation, data collection, digital filtering and dopamine transient detection and analysis. To detect extracellular dopamine a triangle waveform (−0.4 V to 1.3 V vs Ag/AgCl at 400 V/sec) was applied to the carbon-fiber microelectrode at 10 Hz. Changes in extracellular dopamine were determined by monitoring the current over a 100 mV window at the peak oxidation potential for dopamine (about 0.65 V). Dopamine currents *in vivo* were converted to dopamine concentrations by calibrating the electrodes following experimental use with two dopamine standard solutions in a flow injection system. The composition of the buffer used for post-calibration was (in mM): 126 NaCl, 2.5 KCl, 2.4 CaCl_2_, 1.2 MgCl_2_, 2.0 Na_2_SO_4_, 1.2 NaH_2_PO_4_, 15 TRIS HCl, pH = 7.4.

#### In-vivo Methods

Twelve rats were allowed to recover from catheter implantation for at least 3 full days before overnight food and water deprivation. The morning following deprivation, rats were anesthetized with urethane (1.5 g/kg i.p.) and positioned in a stereotaxic apparatus (David Kopf Instruments, Tujunga, CA). Body temperature was maintained at 37°C with a Deltaphase Isothermal Pad (Braintree Scientific, Braintree, MA). The scalp and underlying fascia were resected and holes were drilled for working, stimulating and reference electrodes. The stereotactic coordinates are given in mm anteroposterior (AP) and mediolateral (ML) from bregma, and dorsoventral (DV) from dura. A bipolar stimulating electrode (Plastics One Inc., Roanoke, VA) was positioned in the ventral tegmental area (VTA): −5.2 AP, +1.0 ML, −7.5 to −9.0 DV. A carbon-fiber microelectrode was placed in the ipsilateral nucleus accumbens shell (+1.7 AP, 0.8 ML, −6.4 to −7.4 DV). An Ag/AgCl reference wire was placed in the contralateral cortex. Extracellular dopamine concentrations resulting from 24 pulse 60 Hz stimulation trains (biphasic, 2 ms each phase and 130 µA) of the VTA were recorded. The locations of the stimulating and working electrodes were optimized to find sites that supported electrically-stimulated dopamine release and spontaneous dopamine transients. Dopamine templates for subsequent transient identification were made from cyclic voltammograms obtained from the stimulated release. All sites selected for transient analysis supported stimulated dopamine release with a signal to noise ratio of 30 or more. Tail blood glycemia was determined, as described above, and baseline data were recorded for 6 to 10 minutes without further stimulation. Immediately after the final baseline data collection, the infusion pump was turned on. Glucose (5% in saline) was infused (3 mL/5 min) in either the hepatic portal or jugular vein. For the control group, saline was infused into the portal vein. One minute files were recorded continuously for 20 minutes, after which tail blood glycemia was determined again and maintenance of stimulated release was verified. Glycemia measurement could not be performed in 2 animals, due to glucometer malfunction. To determine if the recorded dopamine release site was responsive to dopamine uptake inhibition, an intraperitoneal injection of 15 mg/kg cocaine was given one hour following infusion. Data were recorded for 25 minutes following cocaine and stimulated release was rechecked. In 4 animals, transient frequency had degraded or was unstable at the time that cocaine was administered.

### Data Analysis

Results from data analyses were expressed as mean ± standard error of the mean and ‘n’ is the number of rats. Analyses were performed with GraphPad Prism (GraphPad Software, Inc., San Diego, California)or NCSS 2000 software (NCSS, Kaysville, UT), and made use of two-way or one-way ANOVAs (with Bonferroni post-hoc tests) and two-sample or one-sample t-tests. Sequential Bonferroni correction for multiple comparisons was performed with the Holm's method [69] whenever multiple independent t-tests were used in the same data set.

#### Behavioral Preference Measures

Two-bottle preference tests were analyzed by calculating the preference ratios as
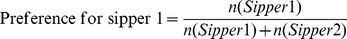
where n(.) denotes the total number of licks detected at a particular sipper during a session. Significance tests were based on one-sample t-tests against 0.5, which is the reference value meaning indifference with respect to either sipper.

#### Blood Glycemia Measures

Baseline glycemia was defined for each blood collection site in each animal by a single measurement performed immediately prior to the initiation of stimulus consumption or administration. Blood glucose levels were analyzed as absolute values (mg/dL) or as % change with respect to baseline. Mean glycemia after stimulus consumption or administration was calculated and the highest glycemia value in this period (peak glycemia) was also identified.

#### Dopamine Transients

Spontaneous dopamine transients were identified using a published procedure that determined the correspondence of putative dopamine peaks to a template voltammogram known to be dopamine [70]. A dopamine cyclic voltammogram that resulted from electrically-stimulated dopamine release served as the template. Baseline cyclic voltammograms were subtracted from those at every individual point using an algorithm in the TH-1 software. For each data file this analysis included four passes using baseline durations of 0.5 to 1.5 seconds that started 0.5, 1.0, 1.5 or 2.5 seconds before the tested data point. The criteria for ascertaining dopamine transients was taken from published methods and included two consecutive points (0.1 second resolution) that had high correspondence with the template (r≥0.86) [14], [15], [70], [71]. A custom-designed Matlab program tabulated the number of transients and their durations (in numbers of 0.1 sec points) and amplitudes by combining results from all the passes. A transient resulted from at least two consecutive points identified as dopamine. Amplitudes were determined as the peak dopamine current of the transient minus the baseline near the onset of the transient peak and converted to dopamine concentration following the *in vitro* post-calibration [14]. The number of consecutive transient points (0.1 sec) was used as transient duration [14].

## Supporting Information

Figure S1 **Consumption of water during conditioning sessions.** Overall consumption of water during conditioning sessions was compared using repeated-measures two-way ANOVA. **A.** Animals conditioned with oral stimuli licked more during vehicle than glucose administration (F = 8.3, p = 0.02), but no effects were found for conditioning stimulus (5% vs. 15% glucose, F = 0.2, p = 0.65) or the interaction between these factors (F = 0.05, p = 0.82). This effect of reduced licking during glucose availability was nevertheless minor, since we found no differences in pair-wise comparisons between consumption in glucose vs. vehicle sessions for animals conditioned with both 5% (4933±117 vs. 5460±141, t = 2.3, p>0.05) and 15% glucose (5136±259 vs. 5585±502, t = 1.8, p>0.05; post-hoc Bonferroni t-tests). **B.** Similar to findings during oral conditioning, animals conditioned with stimuli administered in the JV licked more during vehicle than glucose or saccharin administration (F = 4.5, p = 0.04), but no effects were found for conditioning stimulus (5% glucose vs. 22.5% glucose vs. 50% glucose vs. 3.16% saccharin, F = 1.5, p = 0.23) or the interaction between these factors (F = 1.4, p = 0.27). Again, no differences were found in pair-wise comparisons between consumption in glucose or saccharin vs. vehicle sessions for animals conditioned with 5% glucose (6614±356 vs. 6466±334, t = 0.3, p>0.05), 22.5% glucose (5092±650 vs. 6157±245, t = 1.7,p>0.05), 50% glucose (4824±592 vs. 6062±763, t = 2.5,p>0.05) and 3.16% saccharin (4826±802 vs. 5250±512, t = 0.6, p>0.05; post-hoc Bonferroni t-tests). **C.** In animals with hepatic-portal vein (HPV) catheters, there was overall more licking in those conditioned with glucose relative to the mannitol group (F = 259, p<0.0001), but no effect for vehicle vs. glucose or mannitol administration (F = 0.7, p = 0.41) or the interaction between these factors (F = 0.1, p = 0.73). Furthermore, we found no differences in pair-wise comparisons between consumption in glucose vs. vehicle sessions for animals conditioned with both 5% glucose (7314±148 vs. 7758±139, t = 0.4, p>0.05) and 5% mannitol (3887±183 vs. 4244±574, t = 0.8, p>0.05; post-hoc Bonferroni t-tests).
(TIF)

Figure S2 **Mean and peak tail blood glycemia (mg/dL) measurements in awake animals.** **A.** In animals conditioned orally, differences were not found for mean glycemia after 5% and 15% glucose (84±7.4 vs. 94±3 respectively, t = 1.4, p = 0.23, unpaired t-test). **B.** Mean glycemia after oral 15% glucose, which was effective in conditioning side-bias reversal, was also compared to those resulting from JV administration of glucose. A significant overall effect was found (F = 27.6, p<0.0001, one-way ANOVA) and pair-wise comparisons relative to oral 15% glucose revealed differences for JV 22.5% (220±19.2, t = 3.6, p<0.001) and 50% glucose (315±33.6, t = 7, p<0.001), but not relative to 5% glucose (84±1.8, t = 0.3, p>0.05; post-hoc Bonferroni t-tests). **C.** Mean glycemia after HPV (93±5.9) and JV administration of 5% glucose was not significantly different (t = 1.7, p = 0.13, unpaired t-test). **D–F.** Similar results were found when peak, rather than mean glycemia, was compared. No differences were found between 5% and 15% glucose (97±2.5 vs. 102±4.3 respectively, t = 0.87, p = 0.42, unpaired t-test; D). In comparisons between oral 15% glucose and JV administration of glucose, a significant overall effect was found (F = 34.4, p<0.0001, one-way ANOVA) and pair-wise comparisons revealed differences relative to JV 22.5% (319±26.3, t = 4.9, p<0.001) and 50% glucose (424±42.1, t = 8, p<0.001), but not relative to 5% glucose (110±6.5, t = 0.2, p>0.05; post-hoc Bonferroni t-tests; E). Finally, peak glycemia after HPV (93±5.9) and JV administration of 5% glucose was not significantly different (t = 2, p = 0.08, unpaired t-test, F).
(TIF)

Figure S3 **Mean tail blood glycemia (% baseline) measurements in awake animals.** Glycemia data shown above (Fig. S2), was also analyzed after normalization to baseline (% baseline; also see Figs. 2B, 2C and 3B for peak values) **A.** In animals conditioned orally, differences were not found for mean glycemia after 5% and 15% glucose (100±4.2 vs. 121±7.3, t = 2.3, p = 0.07, unpaired t-test). **B.** Mean glycemia after oral 15% glucose, which was effective in conditioning side-bias reversal, was also compared to those resulting from JV administration of glucose. A significant overall effect was found (F = 20.8, p<0.0001, one-way ANOVA) and pair-wise comparisons relative to oral 15% glucose revealed differences for JV 22.5% (296±29.4, t = 3.6, p<0.01) and 50% glucose (402±46.2, t = 6.3, p<0.001), but not relative to 5% glucose (130±6.4, t = 0.2, p>0.05; post-hoc Bonferroni t-tests). **C.** Mean glycemia after HPV (121±6.6) and JV administration of 5% glucose was not significantly different (t = 1, p = 0.33, unpaired t-test).
(TIF)

Figure S4 **Dopamine transient duration and amplitude in the nucleus accumbens of anesthetized rats, after glucose or vehicle administration in the hepatic-portal or jugular vein.** Fast-scan cyclic voltammetry was used to identify spontaneous dopamine release events (transients) in the nucleus accumbens shell of anesthetized rats. Measurements were conducted for a baseline period, and also during and after infusion of 5% glucose in the JV (n = 4) or HPV (n = 4), or vehicle in the latter (n = 4). Glucose infusion in the HPV, but not the JV, was shown to cause an increase in dopamine transient frequency, when compared to the effect of vehicle infusion in the HPV (see Fig. 5). **A.** Here we show transient duration (msec.) prior to infusion (baseline) and in 5 min bins following infusion onset, for HPV vehicle (baseline, 464±204; 5 minutes,371±117; 10 minutes, 316±29; 15 minutes, 350±74; 20 minutes, 402±82; blue squares), HPV glucose (baseline, 548±79; 5 minutes, 437±48; 10 minutes, 416±34; 15 minutes, 434±61; 20 minutes, 474±61; red squares) and JV glucose (baseline, 534±120; 5 minutes, 507±142; 10 minutes, 464±41; 15 minutes, 396±108; 20 minutes, 493±97; black squares). Two-way ANOVA revealed the absence of any significant effects for treatment (F = 1.6, p = 0.21), time (F = 0.9, p = 0.49) and the interaction between these factors (F = 0.08, p = 1). **B.** For transient amplitude (nM) the values for HPV vehicle (baseline, 35.9±7.4; 5 minutes, 32.8±7.1; 10 minutes, 29.7±7; 15 minutes, 26.5±5.3; 20 minutes, 24.4±3.3), HPV glucose (baseline, 29.3±7.7; 5 minutes, 26.6±4.4; 10 minutes, 30.2±4.8; 15 minutes, 26.5±3.7; 20 minutes, 23.1±3.4) and JV glucose (baseline, 26.3±1.2; 5 minutes, 21.7±1.8; 10 minutes, 24.9±1.8; 15 minutes, 25.9±1.2; 20 minutes, 25.8±3.4) were also compared using two-way ANOVA, and no effects were found for treatment (F = 1.4, p = 0.25), time (F = 0.7, p = 0.58) and the interaction between these factors (F = 0.4, p = 0.91).
(TIF)

Figure S5 **Dopamine transient frequency, duration and amplitude, in the nucleus accumbens of anesthetized rats, after systemic cocaine administration.** To determine if the selected dopamine release site was responsive to dopamine uptake inhibition, once the effects of glucose administration on nucleus accumbens dopamine transients had been measured, 15 mg/kg cocaine was administered through an intraperitoneal injection. In 4 animals, transient frequency had degraded or was unstable. Data for the remaining 8 rats is presented together, irrespective of treatment group. Repeated-measures one-way ANOVA was used to investigate possible changes in transient frequency, duration and amplitude in 5-minute-long time intervals following i.p. injection, when compared to the baseline period immediately prior to cocaine injection. **A.** Cocaine had an overall effect of increasing transient frequency (F = 3.8, p = 0.007) with a significant effect in comparison to baseline (2.2±0.7 transients/min) at 20 minutes (7±2.1, t = 3.3, p<0.05), but not the remaining time points (5 minutes, 2.2±0.7, t = 0.004; 10 minutes, 4.5±1.3, t = 1.5; 15 minutes, 5.9±1.8, t = 2.5; 25 minutes, 5.9±1.6, t = 2.5; p>0.05 for all; post-hoc Bonferroni t-tests). **B.** For transient duration, cocaine also had an overall significant effect (F = 4, p = 0.006), with further significant effects in comparison to baseline (423±32 msec) at 10 minutes (711±122, t = 3.4, p<0.01), 15 minutes (736±126, t = 3.7, p<0.01), 20 minutes (700±65, t = 3.3, p<0.05) and 25 minutes, 705±76, t = 3.4, p<0.01), but not at 5 minutes (600±84, t = 2.1; p>0.05; post-hoc Bonferroni t-tests). **C.** The effects of cocaine on transient amplitude were not significant, both overall (F = 1.3, p = 0.31) and when each time-point was compared to baseline (21.2±2.4 nM; 5 minutes, 23.7±1.9, t = 1.1; 10 minutes, 25.5±3.8, t = 1.9; 15 minutes, 26.5±3.2, t = 2.3; 20 minutes, 23.9±1.8, t = 1.2; 25 minutes, 23.6±2, t = 1.1; p>0.05 for all; post-hoc Bonferroni t-tests).
(TIF)

Table S1 **Tail blood glycemia (mg/dL) measurements in awake animals.** Rats were allowed to drink water with simultaneous administration of one of the glucose solutions used for conditioning (5% or 15% orally, 5%, 22.5% or 50% in the JV or 5% in the HPV). Glycemia was measured from tail blood at the start (baseline) and end (0′) of the 10 minute-long behavioral session, and every 10 minutes thereafter (10′–40′).Glycemia (mg/dL) was compared using repeated-measures two-way ANOVA, revealing significant overall effects for stimulus (JV vs. HPV vs. oral 5% glucose vs. oral 15% glucose vs. JV 22.5% glucose vs. JV 50% glucose; F = 25.4, p<0.0001), time (baseline vs. 0′ vs. 10′ vs. 20′ vs. 30′ vs. 40′; F = 42.24, p<0.0001) and the interaction between these factors (F = 12.5, p<0.0001). Further comparisons were performed at each time-point between glycemia after JV 5% glucose, considered as a control stimulus that did not condition side-bias reversal, and glycemia measured after each of the remaining glucose stimuli (see details in this table). Given the significant interaction, data was also analyzed separately for each stimulus, showing a significant effect for time in all cases (JV 5% glucose, F = 14.48, p<0.0001; JV 22.5% glucose, F = 19.98, p<0.0001; JV 50% glucose, F = 28.04, p<0.0001; oral 15% glucose, F = 3.212, p = 0.036; HPV 5% glucose, F = 26.7, p<0.0001) with the exception of oral 5% glucose (F = 2.68, p = 0.087; repeated-measures one-way ANOVA). Finally, glycemia at each time-point was compared to the respective baseline measure (see details in this table). Significant comparisons are emphasized as bold text. (basel. – baseline).
(DOC)

Table S2 **Blood glycemia (mg/dL) measurements in anesthetized animals.** Rats were anesthetized and then injected with vehicle or one of the different glucose solutions used for conditioning through duodenal (water, n = 5; 5% glucose, n = 4; 15% glucose, n = 6), JV (saline, n = 5; 5% glucose, n = 6; 22.5% glucose, n = 4; 50% glucose, n = 4) or HPV (saline, n = 4; 5% glucose, n = 7) catheters. Glycemia was measured from both tail and HPV blood at the start (baseline) and end (0′) of the stimulus perfusion, and every 10 minutes thereafter (10′–50′). Initially, data were analyzed separately for vehicle and glucose injections and for tail and HPV blood glycemia. **A.** For tail blood glycemia measurements after vehicle injections, a significant overall effect was found for time (baseline vs. 0′ vs. 10′ vs. 20′ vs. 30′ vs. 40′ vs. 50′; F = 2.6, p = 0.02) but not for injection site (JV vs. duodenal vs. HPV; F = 0.3, p = 0.78) or the interaction between these factors (F = 1.1, p = 0.4; repeated-measures two-way ANOVA). Further comparisons were performed at each time-point between glycemia after JV saline and that observed after duodenal water or HPV saline (see details in this table). **B.** With HPV blood glycemia measurements after vehicle injections, significant overall effects were not found for time (F = 1, p = 0.41), injection site (F = 1.3, p = 0.31) nor the interaction between these factors (F = 1.9, p = 0.05; repeated-measures two-way ANOVA). Again, comparisons were performed at each time-point between glycemia after JV saline and that observed after other routes of vehicle administration (see details in this table). **C.** Relative to tail blood glycemia measurements after glucose injections, a significant overall effect was found for time (F = 129.3, p<0.0001), stimulus (JV vs. HPV vs. oral 5% glucose vs. oral 15% glucose vs. JV 22.5% glucose vs. JV 50% glucose; F = 45.5, p<0.0001) and the interaction between these factors (F = 30.7, p<0.0001; repeated-measures two-way ANOVA). Further comparisons were performed at each time-point between glycemia after JV 5% glucose, considered as a control stimulus that did not condition side-bias reversal, and that observed after the remaining glucose stimuli (see details in this table). **D.** In terms of HPV blood glycemia measurements after glucose injections, a significant overall effect was found for time (F = 132, p<0.0001), stimulus (F = 15.5, p<0.0001) and the interaction between these factors (F = 27.5, p<0.0001; repeated-measures two-way ANOVA). Again, further comparisons were performed at each time-point between glycemia measurements after JV 5% glucose and those observed after the remaining glucose stimuli (see details in this table). Significant comparisons are emphasized as bold text. (basel. – baseline; dd. – duodenal; sal. – saline; wat. - water).
(DOC)

Table S3 **Mean and peak blood glycemia (mg/dL) in anesthetized animals.** Tail and HPV blood glycemia measurements in anesthetized rats (see Table S2) were also analyzed according to mean and peak values after glucose or vehicle administration (i.e., 0′–50′). Since, as described above, no overall differences in glycemia were found according to the different routes of vehicle administration, data for vehicle was included as a single category. Glucose stimuli that previously were not shown to be effective in conditioning side-bias reversal are emphasized with bold text. **A.** Mean glycemia was compared using repeated-measures two-way ANOVA, revealing significant overall effects for stimulus (JV vs. HPV vs. duodenal 5% glucose vs. duodenal 15% glucose vs. JV 22.5% glucose vs. JV 50% glucose vs. vehicle; F = 47.56, p<0.0001), blood territory (tail vs. HPV; F = 13.44, p = 0.0007) and the interaction between these factors (F = 25.2, p<0.0001). Significant differences between tail and HPV glycemia were also found for several individual stimuli (see details in this table). Given the significant interaction between factors, tail and HPV glycemia were analyzed separately and, in both cases, overall significant differences were found (respectively: F = 66.26, p<0.0001; F = 31.84, p<0.0001; one-way ANOVA). Further pair-wise comparisons were also performed separately for tail and HPV blood between glycemia measurements after JV 5% glucose, considered as a control stimulus that did not condition side-bias reversal, and those observed after the remaining glucose stimuli. For tail blood measurements, such differences were found for JV 22.5% and 50% glucose and vehicle, while for HPV blood measurements differences were found for duodenal 15% glucose and JV 22.5% and 50% glucose (see details in this table). **B.** Peak glycemia was compared using the same methodologies. We found significant overall effects for stimulus (F = 160.1, p<0.0001), blood territory (F = 32.22, p<0.0001) and the interaction between these factors (F = 10.46, p<0.0001; repeated-measures two-way ANOVA), and also significant differences between tail and HPV glycemia for several stimuli (see table). Again, for both tail and HPV glycemia, overall significant differences were found (respectively: F = 154.8, p<0.0001; F = 93.98, p<0.0001; one-way ANOVA). For tail blood measurements, duodenal 5% glucose, JV 22.5% and 50% glucose and vehicle differed significantly from JV 5% glucose, while for HPV blood measurements, differences were found for vehicle, HPV 5% glucose, duodenal 15% glucose and JV 22.5% and 50% glucose. In the latter case, duodenal 5% glucose, the only other glucose stimulus that did not condition side-bias reversal in awake animals, was the only tested stimulus that did not significantly differ from JV 5% glucose (see details in this table). Significant comparisons are emphasized as bold text. (dd. – duodenal).
(DOC)

Table S4 **Mean and peak blood glycemia (% baseline) in anesthetized animals.** Mean and peak tail and HPV blood glycemia were also compared using values normalized to baseline measures (% of baseline; see Tables S2 and S3). Again, glucose stimuli that previously were not shown to be effective in conditioning side-bias reversal are emphasized with bold text. **A.** For mean relative glycemia we found significant overall effects for stimulus (F = 61.8, p<0.0001), blood territory (F = 10.98, p = 0.002) and the interaction between these factors (F = 21.45, p<0.0001; repeated-measures two-way ANOVA), and significant differences between tail and HPV glycemia were found for several specific stimuli (see details in this table). Given the significant interaction between factors, tail and HPV glycemia were also analyzed separately and overall significant effects were found for both tail blood (F = 78.37, p<0.0001) and HPV blood measurements (F = 39.95, p<0.0001; repeated measures one-way ANOVA). As done previously, further pair-wise comparisons were performed between glycemia measurements after JV 5% glucose, considered as a control stimulus that did not condition side-bias reversal, and those observed after the remaining glucose stimuli. For tail blood, JV 22.5% and 50% glucose were significantly different, while for HPV blood, differences were found for HPV 5% glucose, duodenal 5% and 15% glucose and JV 22.5% and 50% (see table). **B.** For peak relative glycemia, significant main effects were found for stimulus (F = 112.2, p<0.001), blood territory (F = 29.6, p<0.001) and the interaction between these factors (F = 11.4, p<0.001; repeated-measures two-way ANOVA), and also for several specific stimuli between tail and HPV glycemia (see details in this table). Given the significant interaction between factors, tail and HPV glycemia were analyzed separately and, in both cases, significant differences were found (respectively: F = 85.92, p<0.001 and F = 92.8, p<0.001; repeated-measures one-way ANOVA). Furthermore, for tail blood measurements, only JV 22.5% and 50% glucose and vehicle differed significantly from JV 5% glucose, while for HPV blood measurements differences were found for all stimuli except duodenal 5% glucose, the only other glucose stimulus that did not result in side-bias reversal (see details in this table). Significant comparisons are emphasized as bold text. (dd. – duodenal).
(DOC)

Table S5 **Dopamine transient frequency in the nucleus accumbens of anesthetized rats, after glucose or vehicle administration in the hepatic-portal or jugular vein.** Fast-scan cyclic voltammetry was used to identify spontaneous dopamine release events or “transients” in the nucleus accumbens shell of anesthetized rats. Measurements were conducted for a baseline period, and also during and after infusion of 5% glucose in the JV (n = 4) or HPV (n = 4), or vehicle in the latter (n = 4). Glucose infusion in the HPV, but not the JV, was shown to cause an increase in dopamine transient frequency, when compared to the effect of vehicle infusion in the HPV and also the respective baseline values (also see Fig. 5). Significant comparisons are emphasized as bold text. (sal. – saline).
(DOC)
