# Supplementary figures and images for: Intravascular Food Reward
Source: PLoS One. 2011 Sep 27;6(9):e24992. doi: 10.1371/journal.pone.0024992 (PMC3181252; doi:10.1371/journal.pone.0024992)

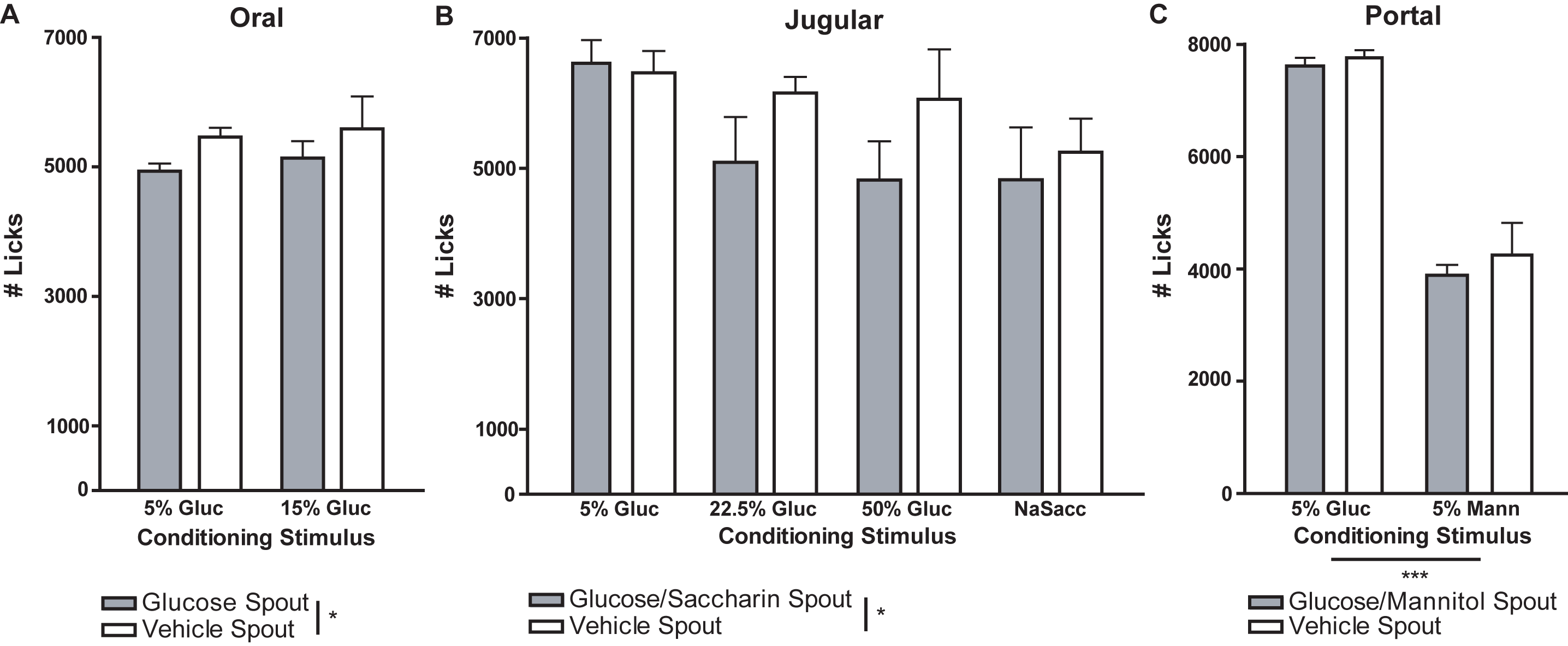

Supplement: Figure S1 — Consumption of water during conditioning sessions. Overall consumption of water during conditioning sessions was compared using repeated-measures two-way ANOVA. A. Animals conditioned with oral stimuli licked more during vehicle than glucose administration (F = 8.3, p = 0.02), but no effects were found for conditioning stimulus (5% vs. 15% glucose, F = 0.2, p = 0.65) or the interaction between these factors (F = 0.05, p = 0.82). This effect of reduced licking during glucose availability was nevertheless minor, since we found no differences in pair-wise comparisons between consumption in glucose vs. vehicle sessions for animals conditioned with both 5% (4933±117 vs. 5460±141, t = 2.3, p>0.05) and 15% glucose (5136±259 vs. 5585±502, t = 1.8, p>0.05; post-hoc Bonferroni t-tests). B. Similar to findings during oral conditioning, animals conditioned with stimuli administered in the JV licked more during vehicle than glucose or saccharin administration (F = 4.5, p = 0.04), but no effects were found for conditioning stimulus (5% glucose vs. 22.5% glucose vs. 50% glucose vs. 3.16% saccharin, F = 1.5, p = 0.23) or the interaction between these factors (F = 1.4, p = 0.27). Again, no differences were found in pair-wise comparisons between consumption in glucose or saccharin vs. vehicle sessions for animals conditioned with 5% glucose (6614±356 vs. 6466±334, t = 0.3, p>0.05), 22.5% glucose (5092±650 vs. 6157±245, t = 1.7,p>0.05), 50% glucose (4824±592 vs. 6062±763, t = 2.5,p>0.05) and 3.16% saccharin (4826±802 vs. 5250±512, t = 0.6, p>0.05; post-hoc Bonferroni t-tests). C. In animals with hepatic-portal vein (HPV) catheters, there was overall more licking in those conditioned with glucose relative to the mannitol group (F = 259, p<0.0001), but no effect for vehicle vs. glucose or mannitol administration (F = 0.7, p = 0.41) or the interaction between these factors (F = 0.1, p = 0.73). Furthermore, we found no differences in pair-wise comparisons between consumption in glucose vs [file pone.0024992.s001.tif]

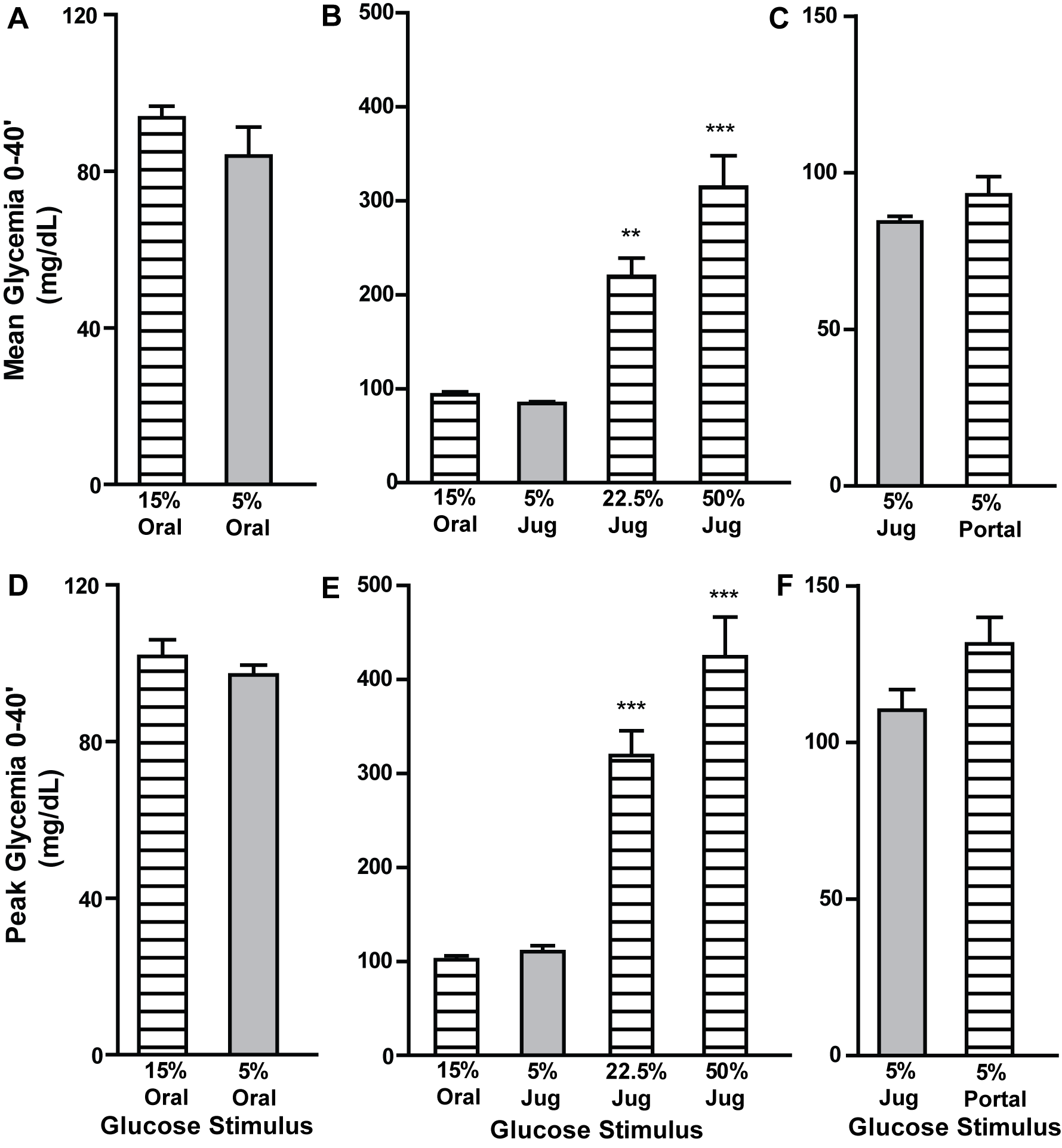

Supplement: Figure S2 — Mean and peak tail blood glycemia (mg/dL) measurements in awake animals. A. In animals conditioned orally, differences were not found for mean glycemia after 5% and 15% glucose (84±7.4 vs. 94±3 respectively, t = 1.4, p = 0.23, unpaired t-test). B. Mean glycemia after oral 15% glucose, which was effective in conditioning side-bias reversal, was also compared to those resulting from JV administration of glucose. A significant overall effect was found (F = 27.6, p<0.0001, one-way ANOVA) and pair-wise comparisons relative to oral 15% glucose revealed differences for JV 22.5% (220±19.2, t = 3.6, p<0.001) and 50% glucose (315±33.6, t = 7, p<0.001), but not relative to 5% glucose (84±1.8, t = 0.3, p>0.05; post-hoc Bonferroni t-tests). C. Mean glycemia after HPV (93±5.9) and JV administration of 5% glucose was not significantly different (t = 1.7, p = 0.13, unpaired t-test). D–F. Similar results were found when peak, rather than mean glycemia, was compared. No differences were found between 5% and 15% glucose (97±2.5 vs. 102±4.3 respectively, t = 0.87, p = 0.42, unpaired t-test; D). In comparisons between oral 15% glucose and JV administration of glucose, a significant overall effect was found (F = 34.4, p<0.0001, one-way ANOVA) and pair-wise comparisons revealed differences relative to JV 22.5% (319±26.3, t = 4.9, p<0.001) and 50% glucose (424±42.1, t = 8, p<0.001), but not relative to 5% glucose (110±6.5, t = 0.2, p>0.05; post-hoc Bonferroni t-tests; E). Finally, peak glycemia after HPV (93±5.9) and JV administration of 5% glucose was not significantly different (t = 2, p = 0.08, unpaired t-test, F). (TIF) [file pone.0024992.s002.tif]

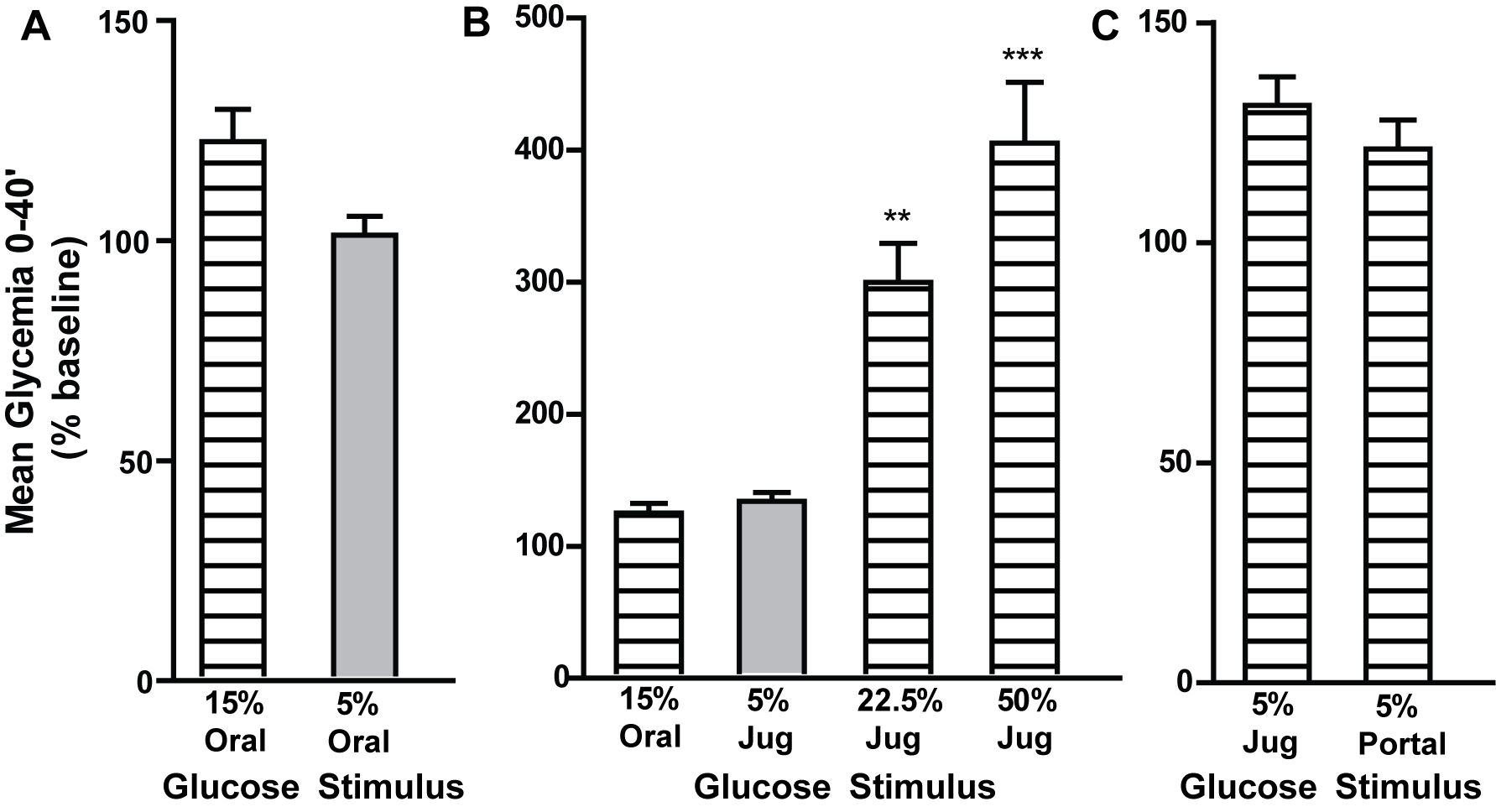

Supplement: Figure S3 — Mean tail blood glycemia (% baseline) measurements in awake animals. Glycemia data shown above (Fig. S2), was also analyzed after normalization to baseline (% baseline; also see Figs. 2B, 2C and 3B for peak values) A. In animals conditioned orally, differences were not found for mean glycemia after 5% and 15% glucose (100±4.2 vs. 121±7.3, t = 2.3, p = 0.07, unpaired t-test). B. Mean glycemia after oral 15% glucose, which was effective in conditioning side-bias reversal, was also compared to those resulting from JV administration of glucose. A significant overall effect was found (F = 20.8, p<0.0001, one-way ANOVA) and pair-wise comparisons relative to oral 15% glucose revealed differences for JV 22.5% (296±29.4, t = 3.6, p<0.01) and 50% glucose (402±46.2, t = 6.3, p<0.001), but not relative to 5% glucose (130±6.4, t = 0.2, p>0.05; post-hoc Bonferroni t-tests). C. Mean glycemia after HPV (121±6.6) and JV administration of 5% glucose was not significantly different (t = 1, p = 0.33, unpaired t-test). (TIF) [file pone.0024992.s003.tif]

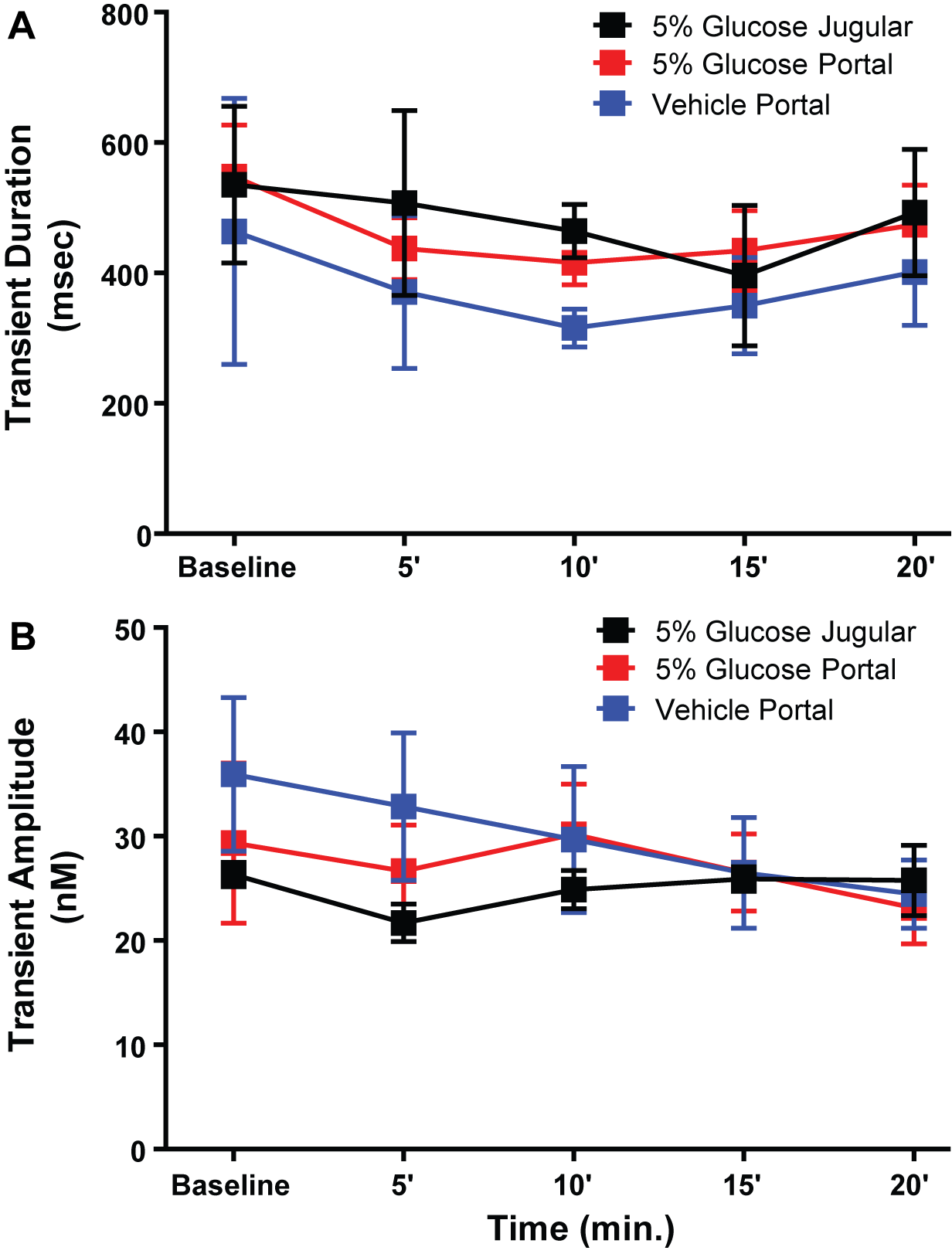

Supplement: Figure S4 — Dopamine transient duration and amplitude in the nucleus accumbens of anesthetized rats, after glucose or vehicle administration in the hepatic-portal or jugular vein. Fast-scan cyclic voltammetry was used to identify spontaneous dopamine release events (transients) in the nucleus accumbens shell of anesthetized rats. Measurements were conducted for a baseline period, and also during and after infusion of 5% glucose in the JV (n = 4) or HPV (n = 4), or vehicle in the latter (n = 4). Glucose infusion in the HPV, but not the JV, was shown to cause an increase in dopamine transient frequency, when compared to the effect of vehicle infusion in the HPV (see Fig. 5). A. Here we show transient duration (msec.) prior to infusion (baseline) and in 5 min bins following infusion onset, for HPV vehicle (baseline, 464±204; 5 minutes,371±117; 10 minutes, 316±29; 15 minutes, 350±74; 20 minutes, 402±82; blue squares), HPV glucose (baseline, 548±79; 5 minutes, 437±48; 10 minutes, 416±34; 15 minutes, 434±61; 20 minutes, 474±61; red squares) and JV glucose (baseline, 534±120; 5 minutes, 507±142; 10 minutes, 464±41; 15 minutes, 396±108; 20 minutes, 493±97; black squares). Two-way ANOVA revealed the absence of any significant effects for treatment (F = 1.6, p = 0.21), time (F = 0.9, p = 0.49) and the interaction between these factors (F = 0.08, p = 1). B. For transient amplitude (nM) the values for HPV vehicle (baseline, 35.9±7.4; 5 minutes, 32.8±7.1; 10 minutes, 29.7±7; 15 minutes, 26.5±5.3; 20 minutes, 24.4±3.3), HPV glucose (baseline, 29.3±7.7; 5 minutes, 26.6±4.4; 10 minutes, 30.2±4.8; 15 minutes, 26.5±3.7; 20 minutes, 23.1±3.4) and JV glucose (baseline, 26.3±1.2; 5 minutes, 21.7±1.8; 10 minutes, 24.9±1.8; 15 minutes, 25.9±1.2; 20 minutes, 25.8±3.4) were also compared using two-way ANOVA, and no effects were found for treatment (F = 1.4, p = 0.25), time (F = 0.7, p = 0.58) and the interaction between these factors (F = 0.4, p = 0.91). (TIF) [file pone.0024992.s004.tif]

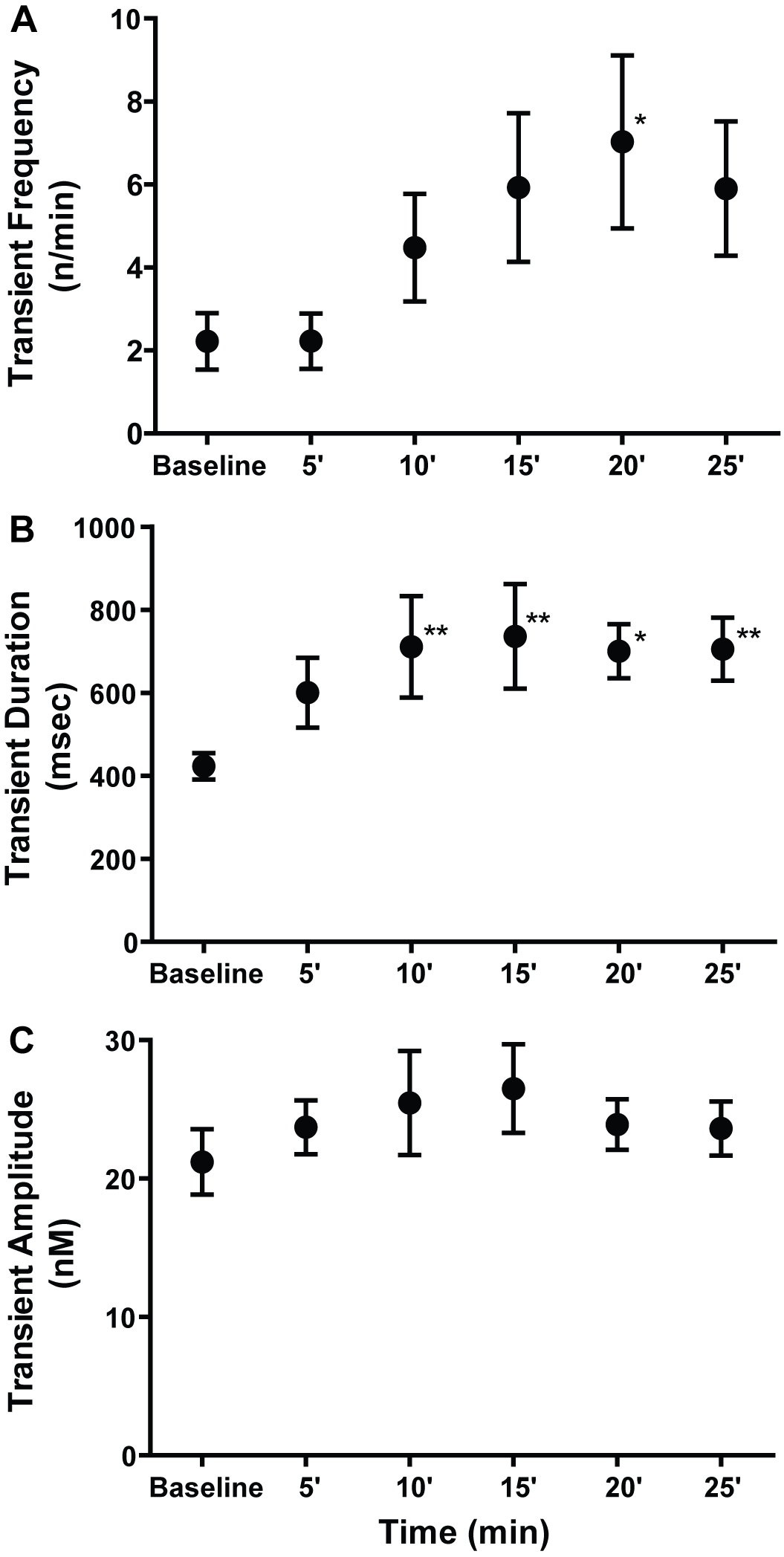

Supplement: Figure S5 — Dopamine transient frequency, duration and amplitude, in the nucleus accumbens of anesthetized rats, after systemic cocaine administration. To determine if the selected dopamine release site was responsive to dopamine uptake inhibition, once the effects of glucose administration on nucleus accumbens dopamine transients had been measured, 15 mg/kg cocaine was administered through an intraperitoneal injection. In 4 animals, transient frequency had degraded or was unstable. Data for the remaining 8 rats is presented together, irrespective of treatment group. Repeated-measures one-way ANOVA was used to investigate possible changes in transient frequency, duration and amplitude in 5-minute-long time intervals following i.p. injection, when compared to the baseline period immediately prior to cocaine injection. A. Cocaine had an overall effect of increasing transient frequency (F = 3.8, p = 0.007) with a significant effect in comparison to baseline (2.2±0.7 transients/min) at 20 minutes (7±2.1, t = 3.3, p<0.05), but not the remaining time points (5 minutes, 2.2±0.7, t = 0.004; 10 minutes, 4.5±1.3, t = 1.5; 15 minutes, 5.9±1.8, t = 2.5; 25 minutes, 5.9±1.6, t = 2.5; p>0.05 for all; post-hoc Bonferroni t-tests). B. For transient duration, cocaine also had an overall significant effect (F = 4, p = 0.006), with further significant effects in comparison to baseline (423±32 msec) at 10 minutes (711±122, t = 3.4, p<0.01), 15 minutes (736±126, t = 3.7, p<0.01), 20 minutes (700±65, t = 3.3, p<0.05) and 25 minutes, 705±76, t = 3.4, p<0.01), but not at 5 minutes (600±84, t = 2.1; p>0.05; post-hoc Bonferroni t-tests). C. The effects of cocaine on transient amplitude were not significant, both overall (F = 1.3, p = 0.31) and when each time-point was compared to baseline (21.2±2.4 nM; 5 minutes, 23.7±1.9, t = 1.1; 10 minutes, 25.5±3.8, t = 1.9; 15 minutes, 26.5±3.2, t = 2.3; 20 minutes, 23.9±1.8, t = 1.2; 25 minutes, 23.6±2, t = 1.1; p>0.05 for all; post-hoc Bonferroni t-tests). (TIF) [file pone.0024992.s005.tif]
