# Supplementary material for: Intravascular Food Reward
Source: PLoS One. 2011 Sep 27;6(9):e24992. doi: 10.1371/journal.pone.0024992 (PMC3181252; doi:10.1371/journal.pone.0024992)
Supplement: Table S1 — Tail blood glycemia (mg/dL) measurements in awake animals. Rats were allowed to drink water with simultaneous administration of one of the glucose solutions used for conditioning (5% or 15% orally, 5%, 22.5% or 50% in the JV or 5% in the HPV). Glycemia was measured from tail blood at the start (baseline) and end (0′) of the 10 minute-long behavioral session, and every 10 minutes thereafter (10′–40′).Glycemia (mg/dL) was compared using repeated-measures two-way ANOVA, revealing significant overall effects for stimulus (JV vs. HPV vs. oral 5% glucose vs. oral 15% glucose vs. JV 22.5% glucose vs. JV 50% glucose; F = 25.4, p<0.0001), time (baseline vs. 0′ vs. 10′ vs. 20′ vs. 30′ vs. 40′; F = 42.24, p<0.0001) and the interaction between these factors (F = 12.5, p<0.0001). Further comparisons were performed at each time-point between glycemia after JV 5% glucose, considered as a control stimulus that did not condition side-bias reversal, and glycemia measured after each of the remaining glucose stimuli (see details in this table). Given the significant interaction, data was also analyzed separately for each stimulus, showing a significant effect for time in all cases (JV 5% glucose, F = 14.48, p<0.0001; JV 22.5% glucose, F = 19.98, p<0.0001; JV 50% glucose, F = 28.04, p<0.0001; oral 15% glucose, F = 3.212, p = 0.036; HPV 5% glucose, F = 26.7, p<0.0001) with the exception of oral 5% glucose (F = 2.68, p = 0.087; repeated-measures one-way ANOVA). Finally, glycemia at each time-point was compared to the respective baseline measure (see details in this table). Significant comparisons are emphasized as bold text. (basel. – baseline). (DOC) [file pone.0024992.s006.doc]

|  | | | Basel. | 0’ | 10’ | 20’ | 30’ | 40’ |
| --- | --- | --- | --- | --- | --- | --- | --- | --- |
| JV 5% | Mean ± SEM | | 65±3 | 110±7 | 89±5 | 80±4 | 79±3 | 64±4 |
|  | vs. basel.* | t | - | 7.1 | 3.7 | 2.4 | 2.2 | 0.3 |
|  |  | p | - | **<0.001** | **<0.01** | >0.05 | >0.05 | >0.05 |
| JV 22.5% | Mean ± SEM | | 75±2 | 300±22 | 279±30 | 224±34 | 162±24 | 135±17 |
|  | vs. basel.* | t | - | 8.1 | 7.4 | 5.4 | 3.1 | 2.2 |
|  |  | p | - | **<0.001** | **<0.001** | **<0.001** | **<0.05** | >0.05 |
|  | vs. JV 5% * | t | 0.3 | 6.2 | 6.2 | 4.7 | 2.7 | 2.3 |
|  |  | p | >0.05 | **<0.001** | **<0.001** | **<0.001** | >0.05 | >0.05 |
| JV 50% | Mean ± SEM | | 80±8 | 415±42 | 401±49 | 323±44 | 247±35 | 186±25 |
|  | vs. basel.* | t | - | 9.6 | 9.2 | 7 | 4.8 | 3 |
|  |  | p | - | **<0.001** | **<0.001** | **<0.001** | **<0.001** | **<0.05** |
|  | vs. JV 5% * | t | 0.5 | 11 | 11.3 | 8.8 | 6.1 | 4.4 |
|  |  | p | >0.05 | **<0.001** | **<0.001** | **<0.001** | **<0.001** | **<0.001** |
| HPV 5% | Mean ± SEM | | 78±8 | 132±9 | 105±4 | 88±11 | 69±2 | 71±7 |
|  | vs. basel.* | t | - | 8.1 | 4.1 | 1.4 | 1.3 | 1.1 |
|  |  | p | - | **<0.001** | **<0.01** | >0.05 | >0.05 | >0.05 |
|  | vs. JV 5% * | t | 0.4 | 0.7 | 0.5 | 0.2 | 0.3 | 0.2 |
|  |  | p | >0.05 | >0.05 | >0.05 | >0.05 | >0.05 | >0.05 |
| Oral 5% | Mean ± SEM | | 84±6 | 86±9 | 96±2 | 84±7 | 76±11 | 78±10 |
|  | vs. basel.* | t | - | 0.3 | 2 | 0 | 1.3 | 0.9 |
|  |  | p | - | >0.05 | >0.05 | >0.05 | >0.05 | >0.05 |
|  | vs. JV 5% * | t | 0.5 | 0.7 | 0.2 | 0.1 | 0.1 | 0.4 |
|  |  | p | >0.05 | >0.05 | >0.05 | >0.05 | >0.05 | >0.05 |
| Oral 15% | Mean ± SEM | | 78±4 | 91±2 | 96±4 | 95±6 | 97±5 | 90±4 |
|  | vs. basel.* | t | - | 2.2 | 3.3 | 3 | 3.4 | 2.1 |
|  |  | p | - | >0.05 | **<0.05** | **<0.05** | **<0.05** | >0.05 |
|  | vs. JV 5% * | t | 0.4 | 0.6 | 0.2 | 0.5 | 0.6 | 0.8 |
|  |  | p | >0.05 | >0.05 | >0.05 | >0.05 | >0.05 | >0.05 |

* post-hoc bonferroni t-tests
