# Supplementary material for: Intravascular Food Reward
Source: PLoS One. 2011 Sep 27;6(9):e24992. doi: 10.1371/journal.pone.0024992 (PMC3181252; doi:10.1371/journal.pone.0024992)
Supplement: Table S2 — Blood glycemia (mg/dL) measurements in anesthetized animals. Rats were anesthetized and then injected with vehicle or one of the different glucose solutions used for conditioning through duodenal (water, n = 5; 5% glucose, n = 4; 15% glucose, n = 6), JV (saline, n = 5; 5% glucose, n = 6; 22.5% glucose, n = 4; 50% glucose, n = 4) or HPV (saline, n = 4; 5% glucose, n = 7) catheters. Glycemia was measured from both tail and HPV blood at the start (baseline) and end (0′) of the stimulus perfusion, and every 10 minutes thereafter (10′–50′). Initially, data were analyzed separately for vehicle and glucose injections and for tail and HPV blood glycemia. A. For tail blood glycemia measurements after vehicle injections, a significant overall effect was found for time (baseline vs. 0′ vs. 10′ vs. 20′ vs. 30′ vs. 40′ vs. 50′; F = 2.6, p = 0.02) but not for injection site (JV vs. duodenal vs. HPV; F = 0.3, p = 0.78) or the interaction between these factors (F = 1.1, p = 0.4; repeated-measures two-way ANOVA). Further comparisons were performed at each time-point between glycemia after JV saline and that observed after duodenal water or HPV saline (see details in this table). B. With HPV blood glycemia measurements after vehicle injections, significant overall effects were not found for time (F = 1, p = 0.41), injection site (F = 1.3, p = 0.31) nor the interaction between these factors (F = 1.9, p = 0.05; repeated-measures two-way ANOVA). Again, comparisons were performed at each time-point between glycemia after JV saline and that observed after other routes of vehicle administration (see details in this table). C. Relative to tail blood glycemia measurements after glucose injections, a significant overall effect was found for time (F = 129.3, p<0.0001), stimulus (JV vs. HPV vs. oral 5% glucose vs. oral 15% glucose vs. JV 22.5% glucose vs. JV 50% glucose; F = 45.5, p<0.0001) and the interaction between these factors (F = 30.7, p<0.0001; repeated-measures two-way ANOVA). Further [file pone.0024992.s007.doc]

| **A) Tail Blood Glycemia** | | | Basel. | 0’ | 10’ | 20’ | 30’ | 40’ | 50’ |
| --- | --- | --- | --- | --- | --- | --- | --- | --- | --- |
| JV sal. | Mean ± SEM | | 105±3 | 99±5 | 92±8 | 94±10 | 90±4 | 93±5 | 88±6 |
| HPV sal. | Mean ± SEM | | 107±6 | 107±6 | 94±8 | 102±4 | 103±9 | 96±6 | 98±7 |
|  | vs.JVsal.* | t | 0.2 | 0.8 | 0.1 | 0.7 | 1.3 | 0.3 | 0.9 |
|  |  | p | >0.05 | >0.05 | >0.05 | >0.05 | >0.05 | >0.05 | >0.05 |
| Dd. wat. | Mean ± SEM | | 94±5 | 100±7 | 99±8 | 99±9 | 96±11 | 97±7 | 92±9 |
|  | vs.JVsal.* | t | 1 | 0.1 | 0.7 | 0.6 | 0.6 | 0.4 | 0.4 |
|  |  | p | >0.05 | >0.05 | >0.05 | >0.05 | >0.05 | >0.05 | >0.05 |
|  | | |  |  |  |  |  |  |  |
| **B) HPV Blood Glycemia** | | | Basel. | 0’ | 10’ | 20’ | 30’ | 40’ | 50’ |
| JV sal. | Mean ± SEM | | 104±3 | 105±5 | 104±7 | 101±7 | 96±9 | 93±8 | 103±8 |
| HPV sal. | Mean ± SEM | | 111±4 | 112±10 | 118±9 | 112±9 | 126±11 | 119±9 | 126±10 |
|  | vs.JVsal.* | t | 0.7 | 0.2 | 0.3 | 0.7 | 0.7 | 1.6 | 0.3 |
|  |  | p | >0.05 | >0.05 | >0.05 | >0.05 | >0.05 | >0.05 | >0.05 |
| Dd. wat. | Mean ± SEM | | 96±6 | 103±9 | 108±8 | 109±10 | 105±10 | 111±11 | 106±7 |
|  | vs.JVsal.* | t | 0.7 | 0.2 | 0.3 | 0.7 | 0.7 | 1.6 | 0.3 |
|  |  | p | >0.05 | >0.05 | >0.05 | >0.05 | >0.05 | >0.05 | >0.05 |
|  | | |  |  |  |  |  |  |  |
| **C) Tail Blood Glycemia** | | | Basel. | 0’ | 10’ | 20’ | 30’ | 40’ | 50’ |
| JV 5% | Mean ± SEM | | 110±7 | 233±11 | 144±11 | 121±8 | 101±9 | 102±10 | 101±13 |
| JV 22.5% | Mean ± SEM | | 109±4.2 | 500±0^#^ | 442±25 | 354±37 | 267±37 | 216±43 | 184±35 |
|  | vs.JV5%* | t | 0.04 | 10.6 | 11.8 | 9.3 | 6.6 | 4.5 | 3.3 |
|  |  | p | >0.05 | **<0.001** | **<0.001** | **<0.001** | **<0.001** | **<0.001** | **<0.001** |
| JV 50% | Mean ± SEM | | 96±11 | 480±20 | 500±0^#^ | 457±17 | 337±39 | 249±35 | 210±41 |
|  | vs.JV5%* | t | 0.5 | 9.8 | 14.1 | 13.3 | 9.4 | 5.9 | 4.3 |
|  |  | p | >0.05 | **<0.001** | **<0.001** | **<0.001** | **<0.001** | **<0.001** | **<0.001** |
| HPV 5% | Mean ± SEM | | 108±5 | 225±18 | 170±17 | 151±17 | 145±14 | 136±15 | 134±15 |
|  | vs.JV5%* | t | 0.07 | 0.4 | 1.2 | 1.4 | 2 | 1.6 | 1.5 |
|  |  | p | >0.05 | >0.05 | >0.05 | >0.05 | >0.05 | >0.05 | >0.05 |
| Dd. 5% | Mean ± SEM | | 87±5 | 103±10 | 123±11 | 132±10 | 129±14 | 125±11 | 114±9 |
|  | vs.JV5%* | t | 0.9 | 5.2 | 0.8 | 0.4 | 1.1 | 0.9 | 0.5 |
|  |  | p | >0.05 | **<0.001** | >0.05 | >0.05 | >0.05 | >0.05 | >0.05 |
| Dd. 15% | Mean ± SEM | | 104±3 | 111±4 | 133±6 | 158±8 | 180±19 | 186±13 | 190±20 |
|  | vs.JV5%* | t | 0.3 | 5.4 | 0.5 | 1.6 | 3.5 | 3.7 | 4 |
|  |  | p | >0.05 | **<0.001** | >0.05 | >0.05 | **<0.01** | **<0.01** | **<0.001** |
|  | | |  |  |  |  |  |  |  |
| **D) HPV Blood Glycemia** | | | Basel. | 0’ | 10’ | 20’ | 30’ | 40’ | 50’ |
| JV 5% | Mean ± SEM | | 117±8 | 220±13 | 136±9 | 111±10 | 107±9 | 104±14 | 94±15 |
| JV 22.5% | Mean ± SEM | | 106±4 | 500±0^#^ | 433±27 | 338±40 | 252±49 | 192±46 | 160±43 |
|  | vs.JV5%* | t | 0.3 | 8.7 | 9.3 | 7.1 | 4.5 | 2.8 | 2.1 |
|  |  | p | >0.05 | **<0.001** | **<0.001** | **<0.001** | **<0.001** | **<0.05** | >0.05 |
| JV 50% | Mean ± SEM | | 96±7 | 500±0^#^ | 500±0^#^ | 372±47 | 252±57 | 165±35 | 102±21 |
|  | vs.JV5%* | t | 0.6 | 8.7 | 11.4 | 8.2 | 4.5 | 1.9 | 0.2 |
|  |  | p | >0.05 | **<0.001** | **<0.001** | **<0.001** | **<0.001** | >0.05 | >0.05 |
| HPV 5% | Mean ± SEM | | 107±6 | 289±16 | 189±22 | 162±22 | 152±21 | 150±15 | 152±17 |
|  | vs.JV5%* | t | 0.3 | 2.5 | 1.9 | 1.9 | 1.6 | 1.7 | 2.1 |
|  |  | p | >0.05 | >0.05 | >0.05 | >0.05 | >0.05 | >0.05 | >0.05 |
| Dd. 5% | Mean ± SEM | | 86±4 | 167±34 | 175±17 | 168±16 | 153±14 | 142±12 | 129±12 |
|  | vs.JV5%* | t | 0.9 | 1.6 | 1.2 | 1.8 | 1.4 | 1.2 | 1.1 |
|  |  | p | >0.05 | >0.05 | >0.05 | >0.05 | >0.05 | >0.05 | >0.05 |
| Dd. 15% | Mean ± SEM | | 102±9 | 177±17 | 226±14 | 289±28 | 296±23 | 296±22 | 293±28 |
|  | vs.JV5%* | t | 0.5 | 1.5 | 3.1 | 6.2 | 6.6 | 6.7 | 7 |
|  |  | p | >0.05 | >0.05 | **<0.05** | **<0.001** | **<0.001** | **<0.001** | **<0.001** |

* post-hoc bonferroni t-tests

^#^ these values reflect the upper detection limit of the glucometer used for this experiment.
