# Supplementary material for: Intravascular Food Reward
Source: PLoS One. 2011 Sep 27;6(9):e24992. doi: 10.1371/journal.pone.0024992 (PMC3181252; doi:10.1371/journal.pone.0024992)
Supplement: Table S3 — Mean and peak blood glycemia (mg/dL) in anesthetized animals. Tail and HPV blood glycemia measurements in anesthetized rats (see Table S2) were also analyzed according to mean and peak values after glucose or vehicle administration (i.e., 0′–50′). Since, as described above, no overall differences in glycemia were found according to the different routes of vehicle administration, data for vehicle was included as a single category. Glucose stimuli that previously were not shown to be effective in conditioning side-bias reversal are emphasized with bold text. A. Mean glycemia was compared using repeated-measures two-way ANOVA, revealing significant overall effects for stimulus (JV vs. HPV vs. duodenal 5% glucose vs. duodenal 15% glucose vs. JV 22.5% glucose vs. JV 50% glucose vs. vehicle; F = 47.56, p<0.0001), blood territory (tail vs. HPV; F = 13.44, p = 0.0007) and the interaction between these factors (F = 25.2, p<0.0001). Significant differences between tail and HPV glycemia were also found for several individual stimuli (see details in this table). Given the significant interaction between factors, tail and HPV glycemia were analyzed separately and, in both cases, overall significant differences were found (respectively: F = 66.26, p<0.0001; F = 31.84, p<0.0001; one-way ANOVA). Further pair-wise comparisons were also performed separately for tail and HPV blood between glycemia measurements after JV 5% glucose, considered as a control stimulus that did not condition side-bias reversal, and those observed after the remaining glucose stimuli. For tail blood measurements, such differences were found for JV 22.5% and 50% glucose and vehicle, while for HPV blood measurements differences were found for duodenal 15% glucose and JV 22.5% and 50% glucose (see details in this table). B. Peak glycemia was compared using the same methodologies. We found significant overall effects for stimulus (F = 160.1, p<0.0001), blood territory (F = 32.22, p<0.0001) and the interaction bet [file pone.0024992.s008.doc]

|  | | | 1. Mean Glycemia (mg/dL) | | | | 1. Peak Glycemia (mg/dL) | | | |
| --- | --- | --- | --- | --- | --- | --- | --- | --- | --- | --- |
|  | | | blood | | Tail vs. HPV* | | blood | | Tail vs. HPV* | |
|  | | | Tail | HPV | t | p | Tail | HPV | t | p |
| **JV 5%** | Mean ± SEM | | 145±14 | 138±13 | 0.8 | >0.05 | 244±14 | 230±15 | 1 | >0.05 |
| JV 22.5% | Mean ± SEM | | 327±28 | 312±30 | 1.3 | >0.05 | 500±0^#^ | 500±0^#^ | 0 | >0.05 |
|  | vs.JV5%* | t | 9.7 | 7.4 | - | - | 13.6 | 11.5 | - | - |
|  |  | p | **<0.001** | **<0.001** | - | - | **<0.001** | **<0.001** | - | - |
| JV 50% | Mean ± SEM | | 372±16 | 315±26 | 5.1 | **<0.001** | 500±0^#^ | 500±0^#^ | 0 | >0.05 |
|  | vs.JV5%* | t | 12.1 | 7.5 | - | - | 13.6 | 11.5 | - | - |
|  |  | p | **<0.001** | **<0.001** | - | - | **<0.001** | **<0.001** | - | - |
| HPV 5% | Mean ± SEM | | 160±14 | 182±18 | 2.6 | >0.05 | 226±18 | 289±16 | 4.6 | **<0.001** |
|  | vs.JV5%* | t | 0.9 | 2.2 | - | - | 1.1 | 2.9 | - | - |
|  |  | p | >0.05 | >0.05 | - | - | >0.05 | **<0.05** | - | - |
| **Dd. 5%** | Mean ± SEM | | 121±10 | 156±17 | 3.1 | **<0.05** | 140±12 | 188±25 | 2.7 | >0.05 |
|  | vs.JV5%* | t | 1.3 | 0.7 | - | - | 5.5 | 1.8 | - | - |
|  |  | p | >0.05 | >0.05 | - | - | **<0.001** | >0.05 | - | - |
| Dd. 15% | Mean ± SEM | | 160±10 | 263±18 | 11.2 | **<0.001** | 201±17 | 321±28 | 8.2 | **<0.001** |
|  | vs.JV5%* | t | 0.9 | 6 | - | - | 2.6 | 4.4 | - | - |
|  |  | p | >0.05 | **<0.001** | - | - | >0.05 | **<0.001** | - | - |
| Vehicle | Mean ± SEM | | 96±4 | 108±5 | 1.9 | >0.05 | 106±4 | 120±5 | 1.4 | >0.05 |
|  | vs.JV5%* | t | 3.5 | 1.7 | - | - | 9.9 | 6.4 | - | - |
|  |  | p | **<0.001** | >0.05 | - | - | **<0.001** | **<0.001** | - | - |

* post-hoc bonferroni t-tests

^#^ these values reflect the upper detection limit of the glucometer used for this experiment.
