# Supplementary material for: Intravascular Food Reward
Source: PLoS One. 2011 Sep 27;6(9):e24992. doi: 10.1371/journal.pone.0024992 (PMC3181252; doi:10.1371/journal.pone.0024992)
Supplement: Table S4 — Mean and peak blood glycemia (% baseline) in anesthetized animals. Mean and peak tail and HPV blood glycemia were also compared using values normalized to baseline measures (% of baseline; see Tables S2 and S3). Again, glucose stimuli that previously were not shown to be effective in conditioning side-bias reversal are emphasized with bold text. A. For mean relative glycemia we found significant overall effects for stimulus (F = 61.8, p<0.0001), blood territory (F = 10.98, p = 0.002) and the interaction between these factors (F = 21.45, p<0.0001; repeated-measures two-way ANOVA), and significant differences between tail and HPV glycemia were found for several specific stimuli (see details in this table). Given the significant interaction between factors, tail and HPV glycemia were also analyzed separately and overall significant effects were found for both tail blood (F = 78.37, p<0.0001) and HPV blood measurements (F = 39.95, p<0.0001; repeated measures one-way ANOVA). As done previously, further pair-wise comparisons were performed between glycemia measurements after JV 5% glucose, considered as a control stimulus that did not condition side-bias reversal, and those observed after the remaining glucose stimuli. For tail blood, JV 22.5% and 50% glucose were significantly different, while for HPV blood, differences were found for HPV 5% glucose, duodenal 5% and 15% glucose and JV 22.5% and 50% (see table). B. For peak relative glycemia, significant main effects were found for stimulus (F = 112.2, p<0.001), blood territory (F = 29.6, p<0.001) and the interaction between these factors (F = 11.4, p<0.001; repeated-measures two-way ANOVA), and also for several specific stimuli between tail and HPV glycemia (see details in this table). Given the significant interaction between factors, tail and HPV glycemia were analyzed separately and, in both cases, significant differences were found (respectively: F = 85.92, p<0.001 and F = 92.8, p<0.001; repeated-measures one-way ANO [file pone.0024992.s009.doc]

|  | | | 1. Mean Glycemia (% baseline) | | | | 1. Peak Glycemia (% baseline) | | | |
| --- | --- | --- | --- | --- | --- | --- | --- | --- | --- | --- |
|  | | | blood | | Tail vs. HPV* | | blood | | Tail vs. HPV* | |
|  | | | Tail | HPV | t | p | Tail | HPV | t | p |
| **JV 5%** | Mean ± SEM | | 124±11 | 112±11 | 1.2 | >0.05 | 215±13 | 192±14 | 1.5 | >0.05 |
| JV 22.5% | Mean ± SEM | | 299±14 | 293±20 | 0.5 | >0.05 | 462±17 | 473±20 | 0.6 | >0.05 |
|  | vs.JV5%* | t | 9.6 | 8 | - | - | 9.4 | 11.39 | - | - |
|  |  | p | **<0.001** | **<0.001** | - | - | **<0.001** | **<0.001** | - | - |
| JV 50% | Mean ± SEM | | 399±34 | 335±42 | 4.9 | **<0.001** | 541±58 | 528±36 | 0.7 | >0.05 |
|  | vs.JV5%* | t | 15.1 | 9.9 | - | - | 12.4 | 13.6 | - | - |
|  |  | p | **<0.001** | **<0.001** | - | - | **<0.001** | **<0.001** | - | - |
| HPV 5% | Mean ± SEM | | 148±13 | 171±15 | 2.3 | >0.05 | 206±13 | 274±20 | 4.7 | **<0.001** |
|  | vs.JV5%* | t | 1.5 | 3 | - | - | 0.4 | 3.9 | - | - |
|  |  | p | >0.05 | **<0.05** | - | - | >0.05 | **<0.01** | - | - |
| **Dd. 5%** | Mean ± SEM | | 139±5 | 179±11 | 3.1 | **<0.05** | 161±5 | 216±17 | 2.9 | **<0.05** |
|  | vs.JV5%* | t | 0.8 | 3 | - | - | 2.1 | 1 | - | - |
|  |  | p | >0.05 | **<0.05** | - | - | >0.05 | >0.05 | - | - |
| Dd. 15% | Mean ± SEM | | 153±7 | 262±12 | 10.3 | **<0.001** | 193±13 | 318±16 | 8.1 | **<0.001** |
|  | vs.JV5%* | t | 1.8 | 7.4 | - | - | 1 | 5.7 | - | - |
|  |  | p | >0.05 | **<0.001** | - | - | >0.05 | **<0.001** | - | - |
| Vehicle | Mean ± SEM | | 95±3 | 105±4 | 1.4 | >0.05 | 105±3 | 116±4 | 1.1 | >0.05 |
|  | vs.JV5%* | t | 2.1 | 0.4 | - | - | 5.6 | 4.1 | - | - |
|  |  | p | >0.05 | >0.05 | - | - | **<0.001** | **<0.01** | - | - |

* post-hoc bonferroni t-tests
