# Supplementary material for: Intravascular Food Reward
Source: PLoS One. 2011 Sep 27;6(9):e24992. doi: 10.1371/journal.pone.0024992 (PMC3181252; doi:10.1371/journal.pone.0024992)
Supplement: Table S5 — Dopamine transient frequency in the nucleus accumbens of anesthetized rats, after glucose or vehicle administration in the hepatic-portal or jugular vein. Fast-scan cyclic voltammetry was used to identify spontaneous dopamine release events or “transients” in the nucleus accumbens shell of anesthetized rats. Measurements were conducted for a baseline period, and also during and after infusion of 5% glucose in the JV (n = 4) or HPV (n = 4), or vehicle in the latter (n = 4). Glucose infusion in the HPV, but not the JV, was shown to cause an increase in dopamine transient frequency, when compared to the effect of vehicle infusion in the HPV and also the respective baseline values (also see Fig. 5). Significant comparisons are emphasized as bold text. (sal. – saline). (DOC) [file pone.0024992.s010.doc]

| Transient Frequency (n/minute) | | | Baseline | 5’ | 10’ | 15’ | 20’ |
| --- | --- | --- | --- | --- | --- | --- | --- |
| HPV saline | Mean ± SEM | | 1.9±1.1 | 1.7±0.7 | 2.2±0.8 | 2.7±1.4 | 1.9±1.6 |
|  | vs. baseline* | t | - | 0.2 | 0.4 | 1 | 0.03 |
|  |  | p | - | >0.05 | >0.05 | >0.05 | >0.05 |
| HPV 5% | Mean ± SEM | | 3.9±0.5 | 8.8±2.8 | 6.6±1.9 | 5.9±1.1 | 3.1±1.1 |
|  | vs. baseline* | t | - | 3 | 1.6 | 1.2 | 0.5 |
|  |  | p | - | **<0.05** | >0.05 | >0.05 | >0.05 |
|  | vs. HPV sal.* | t | 0.9 | 3.1 | 1.9 | 1.4 | 0.5 |
|  |  | p | >0.05 | **<0.05** | >0.05 | >0.05 | >0.05 |
| JV 5% | Mean ± SEM | | 4.4±1.5 | 3.7±2 | 4.1±2.3 | 3.3±2.1 | 2.8±1.9 |
|  | vs. baseline* | t | - | 0.6 | 0.3 | 1.1 | 1.6 |
|  |  | p | - | >0.05 | >0.05 | >0.05 | >0.05 |
|  | vs. HPV sal.* | t | 1.1 | 0.8 | 0.8 | 0.3 | 0.4 |
|  |  | p | >0.05 | >0.05 | >0.05 | >0.05 | >0.05 |

* post-hoc bonferroni t-tests
